# Supplementary material for: Pathobiology of Candida auris infection analyzed by multiplexed imaging and single cell analysis
Source: PLoS One. 2024 Jan 17;19(1):e0293011. doi: 10.1371/journal.pone.0293011 (PMC10793899; doi:10.1371/journal.pone.0293011)
Supplement: S1 File — Protocol document from protocol io (dx.doi.org/10.17504/protocols.io.bpyxmpxn), which outlines antibody characterization for multiplexing procedures. (PDF) [file pone.0293011.s001.pdf]

Cell DIVE™ Platform | Antibody Characterization for Multiplexing

Liz McDonough[1], Chrystal Chadwick[1], Fiona Ginty[1], Christine Surrette[1], Anup Sood[1]

[1]GE Research

Human BioMolecular Atlas Program (HuBMAP) Method Development Community GE F

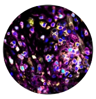

Liz McDonough
GE Research

DEC 16, 2020

OPEN ACCESS

DOI:
dx.doi.org/10.17504/protocols.io.bpyxmpxn

Protocol Citation: Liz McDonough, Chrystal Chadwick, Fiona Ginty, Christine Surrette, Anup Sood 2020. Cell DIVE™ Platform | Antibody Characterization for Multiplexing. **protocols.io** https://dx.doi.org/10.17504/protocols.io.bpyxmpxn

License: This is an open access protocol distributed under the terms of the Creative Commons Attribution License, which permits unrestricted use, distribution, and reproduction in any medium, provided the original author and source are credited

Protocol status: Working
We use this protocol and it's working

Created: Nov 23, 2020

Last Modified: Dec 16, 2020

PROTOCOL integer ID: 44791

Keywords: Antibody, Cell DIVE, Validation, zenon label, multiplexing, characterization

ABSTRACT

This protocol describes the process of validating antibodies (primary/secondary, direct conjugates, and zenon labelled) as per the Cell DIVE™ technology. It also describes the process for determining any antigen effects from the dye inactivation process.

ATTACHMENTS

_Cell_DIVE-manual-Ab-validation_final-version.pdf

GUIDELINES

A standardized antibody characterization process includes selection of multiple clones (initially 2-3, but in some cases more) based on literature reports of their use in IHC and evaluation of their performance using appropriate positive and negative controls using a labeled secondary antibody for detection.

The best performing clone is then conjugated to a fluorescent dye at two dye/protein (D:P) ratios and retested on the same TMA at multiple concentrations to compare sensitivity and specificity with the unmodified primary antibody.

Occasionally antibody conjugates fail to perform. In such cases, these can be stained with secondary detection but their number is limited as they need to be raised in different species or belong to different IgG subtypes to prevent cross reactivity. When multiplexing, an IgG blocking step must be performed before subsequently staining a direct conjugate of the same isotype.

To test antigen effects, or epitope stability to the Cell DIVE cycling process, unstained slides are processed through multiple rounds (typically 0, 1, 5 & 10) of signal inactivation process and then stained to evaluate target expression.

MATERIALS

Required Materials

A	B
Material	Definitions

A	B
0.45µm filter	Filtration of mounting media
Amber glass bottles	Storage for mounting media and/or DAPI
Amber 1.5mL Eppendorf tubes	Storage for antibody direct conjugates
15mL conical tubes	Storage for mixing solutions
50mL conical tubes	Storage for Antibody diluent
Weigh Boats	Used to weigh out solid/powder reagents on analytical balance
Coverslips	Used to coverslip the tissue slides
Eppendorf 1.5mL tubes	Container for antibody dilution preparations
Pipette Tips (0.5-1000µL)	Used for pipettes
Serological Pipettes	Deliver liquid volumes (mL) for solutions
Transfer Pipettes	Delivers PBS for decoverslipping
Lab Tape	Labels bottles and tally scoring
Xylene-Resistant slide labels	Labels printed on the Zebra printer which are resistant to xylene
Nitrile Gloves	Personal Protective Equipment
Kimwipes (large and small)	Clean mounting media from slides
Blue Underpads	Underpad used to absorb reagent spills on the work bench

Required Reagents and Stock Solutions

A	B
DAPI Stock Solution	10 mg vial of DAPI dilactate<br>2.0 mL ddH20<br>----------------------<br>2 mL Total Volume<br><br>Mix thoroughly until solid is dissolved.<br><br>Aliquot into (20) 100 uL aliquots into amber eppendorfs.<br><br>Store in -20 deg C.
0.5 M NaHCO3 , pH 11.2 (acceptable range 10.9-11.3)	42 g NaHCO3<br>1000 mL ddH20<br>----------------------<br>1000 mL Total Volume<br><br>Mix thoroughly until solid is dissolved thoroughly and pH ~11.2 (~15-18g of NaOH pellets).<br><br>The acceptable range for pH is between 10.9 and 11.3. Check and log the pH on a weekly basis during periods of use. If the pH is measured outside of this specification, discard the buffer and do not use.<br><br>Store at 4C for up to 6 months.

A	B
Jackson Immuno Cy-Dye Secondary Antibodies	Secondary antibodies should be reconstituted in ddH20 to a final concentration of 1mg/mL. Make 5ul aliquot in amber tubes and store at -20C up to 6 months.
R&D Systems isotype Controls	Mouse IgG2a, IgG2b and Rat IgG1, IgG2a, and IgG2b isotype controls should be reconstituted in 1mL of 1X PBS. This should yield a final concentration of 1mg/ml or 0.5mg/ml depending on the amount supplied.
Cell Signaling Isotype Controls	Ms IgG1 and Rabbit IgG1 isotype controls are ready to use. Store at -20C.
Lambda Protein Phosphatase Kit	New England Biolabs, Cat # P0753S
Immunizing Peptide	This is antibody specific, and therefore vendor specific. Please contact the vendor for the Ab you are using to determine if they have immunizing peptides available for that antibody.

Table 1: Summary of the required stock solutions and reagents for the workflow.

The below table contains the respective expiration and storage condition for each stock reagent or solution.

A	B	C
Solution	Expiration	Storage
Liquid Chemicals		
Ethanol	2 years from receipt	RT-flammable cabinet
DABCO 4mM	1 week	4 degrees
Phosphate buffered saline	1 year from receipt	RT
30% Hydrogen Peroxide	6 months from receipt	4 degrees C
DAPI	1 year from receipt	-20 degrees C
Glycerol	--	RT
Dry Chemicals		
Propyl gallate	2 years from receipt	RT
DABCO	2 years from receipt	4 degrees C
BSA	2 years from receipt	4 degrees C
Donkey Serum	1 year from receipt	-20 degrees C
Sodium bicarbonate	2 years from receipt	RT
Sodium Hydroxide Pellets	2 years from receipt	RT

Table 2: Summary of the required reagents and stock solutions, and their respective shelf time and storage conditions.

Required Equipment

A	B
Equipment	Definitions
Timer	Used to for timed reactions and/or processes
Humidified Chamber	Used for phosphatase pretreatment
Pipet boy	Used for serological pipettes
Pipettes (2-1000µL)	Deliver liquid volumes (µL) for dilutions and solutions
Graduated Cylinders (25mL,100mL, 250, mL 500 mL, 1000mL)	Used to measure solution volumes
Staining dish	Slide reagent container used during incubations
Slide racks	Rack to hold slides in place during incubations
Orbital Shaker	Decoverslipping process and incubations
Analytical balance	Weighing out reagents
Stir plate	Mixing solutions
Microcentrifuge	Spinning down solutions in tubes
Vortexer	Mix solutions in tubes

Table 3: Summary table of the required equipment and how each is used in the workflow.

SAFETY WARNINGS

Warning: For research use only.

Cell DIVE software and workflows are for internal research use only and not for third party service use or clinical diagnosis.

All chemicals should be considered as potentially hazardous. We therefore recommend that this product is handled only by those persons who have been trained in laboratory techniques and that it is used in accordance with the principles of good laboratory practice. Wear suitable protective clothing such as laboratory coats, safety glasses, and gloves. Care should be taken to avoid contact with skin or eyes. In the case of contact with skin or eyes wash immediately with water.

For hazard information and safety warnings, please refer to the SDS (Safety Data Sheet).

BEFORE START INSTRUCTIONS

Reagent Prep

Working Solutions

A	B
Working Solution	Expiration
1X Phosphate Buffered Saline (PBS)	100 mL 10x PBS<br>900mL ddH20<br>---------------------<br>1000mL Total Volume<br><br>Mix thoroughly
Ab Diluent (3% BSA in PBS)	50 mL 1X PBS<br>1.5 g BSA<br>---------------------<br>50 mL Total Volume<br><br>Mix thoroughly and vortex in a 50 mL tube until all particulates are in solution. Store at 4C for no longer than a week.
DAPI Staining Solution	0.1 mL DAPI stock solution<br>499.9 mL 1X PBS<br>---------------------<br>500 mL Total Volume<br><br>Add 250 mL to 2 opaque staining dishes.<br>Discard after 10 uses. Working solution expires in 6 months from preparation. Store the solution in 4C.
Mounting Media	10 mL 1X PBS<br>90 mL glycerol<br>4.0 g Propyl Gallate<br>1.0 g DABCO<br>---------------------<br>100 mL Total Volume<br><br>Mix contents in a glass bottle and heat overnight in water bath at 60C. Keep protected from light.<br><br>The next day make sure that all contents are in solution and filter with a 0.45uM filter. Cover with foil and store at 4C for up to 2 months.<br><br>Alternate mounting media (50% glycerol, 4% propyl gallate) should be used with markers that leach.
Dye Inactivation Solution	If using white coplin jars (hold ~50mL)<br>10 mL 0.5 M NaHCO3 , pH 11.2 (acceptable range 10.9-11.3)<br>35 mL ddH2O<br>5 mL 30% H2O2<br>-----------<br>50 mL total volume<br><br>If using green staining jars (hold ~250mL)<br>50 mL 0.5 M NaHCO3 , pH 11.2 (acceptable range 10.9-11.3)<br>175 mL ddH2O<br>25 mL 30% H2O2<br>-----------<br>250 mL total volume<br><br>Mix ddH2O and 0.5M NaHCO3 (pH 11.2) thoroughly. This makes up Part 1 of the solution. The 30% H2O2 is Part 2 of the solution.<br>Immediately before putting the slides in the solution, add Part 2 to Part 1 and mix thoroughly.

Table 5: Summary of the required working solutions for the workflow.

Critical Parameters

Please take particular note of the following instructions regarding critical steps:

- It is essential to read the complete instruction booklet before starting work.
- These instructions have only been validated on formalin-fixed paraffin embedded tissue sections.
- Unless noted, it is essential to allow reagents discussed to reach room temperature prior to use.
- Mix samples and all reagents thoroughly before use.
- The pH of the 0.5 M $NaHCO_3$ must be between pH 10.9 and pH 11.3.
- Avoid extensive exposure of fluorescent reagents to ambient light.

Background Imaging

1 Refer to Cell DIVE™ Imaging Manual.

Decoverslipping

2h 10m

2 Take a plastic slide box and place it in secondary containment.

3 Fill the plastic slide box with 1 x PBS .

4 Rest the coverslipped slides on the interior raised edges of slide box such that they are inverted (ie. coverslip down and barcode up).

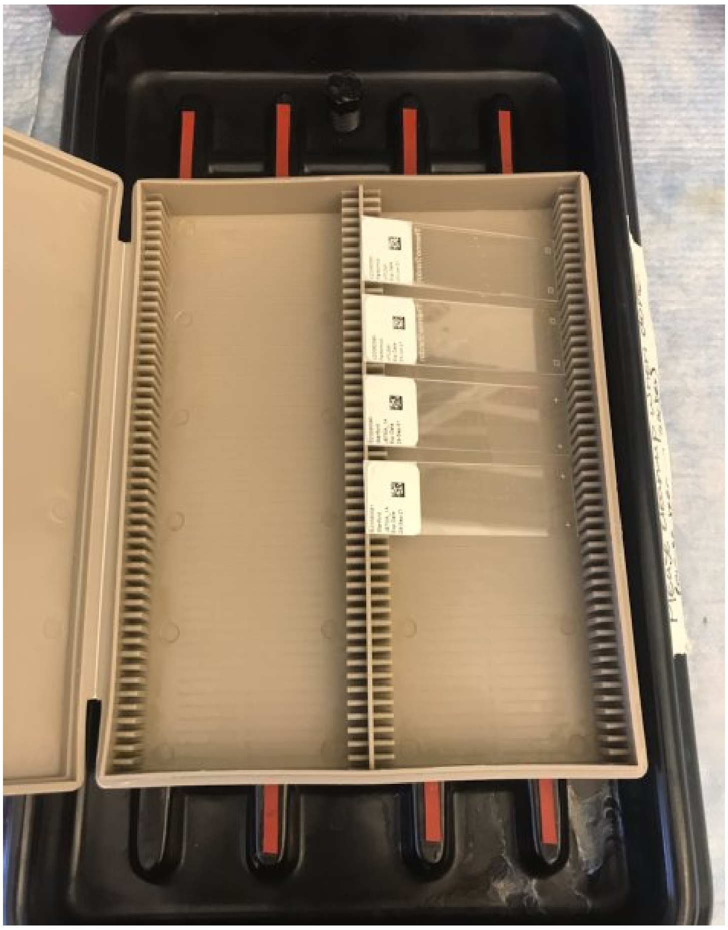

5 If your next round is a staining round, prepare your dilutions while you are waiting for the coverslips to come off.

6 Be patient - within 00:30:00 - 01:00:00 , coverslips should naturally come off. 1h 30m

Calculating Ab Amount for Ab Dilution Cocktails

2h 10m

7 The following section should be used when making cocktails of antibodies for any type of experiment including primary/secondary (P/S), antigen effects (AE), direct conjugates (DC), and revalidation (RV). This includes running 2 different P/S antibodies or conjugates with 2 different dyes on the same slide at the same time. For P/S antibodies, the species (Rabbit IgG, Mouse IgG1, etc) must be different to run on the same slide at the same time.

8 Determine the total volume that will be needed for the experiment.

8.1 For manual workflow, this is typically (200µL*#of slides)

9 Determine the stock concentration for the antibodies. This should be written on the barcode or label on the antibody. If there is no concentration written on the vial or the datasheet contact either the vendor and/or chemist who prepared it and ask. Provide the catalog number and lot#, if applicable.

10 Once a concentration is obtained for an antibody, determine the final concentration the antibodies or conjugates should be run at.

- If the antibody/DC is new and commercially available and no final concentration is noted on the vendor datasheet, call the vendor to ask. If no final concentration can be recommended, run the new marker at [M] 5 microgram per milliliter (µg/mL)
- If the Ab is a GE Validated DC, refer to the DC catalog to determine the optimal working concentration.

11 Use the following formula to calculate the required amount for each antibody (Ab):

- stock concentration * X = final concentration Ab1 * total volume needed for experiment
- stock concentration * X = final concentration Ab2 * total volume needed for experiment
- Solve for X in each case to determine how many µL of each antibody/DC will be required.
- Subtract the sum of the Ab volumes together from the total volume to get the volume of Ab diluent (3% BSA in PBS) that needs to be added.

Note

For example: if 20 µL Ab1 and 40 µL Ab2 was used, the sum of Ab volumes= 60 µL . Subtract this volume from the total volume needed for the experiment to obtain the volume of Ab diluent to add to the Abs.

12 If you are using secondary antibodies, determine the volume you will need for the secondary in the same manner described above for calculating for the primary antibody/DC. Secondary antibodies are stained at a final concentration of [M] 5 microgram per milliliter (µg/mL) . Determine which secondaries you need based on the isotypes of the P/S antibodies used.

13 Determine if there is a dye preference for each marker (ie. Some markers look better in Cy3 or Cy5). If there is no preference, make the marker that has lower expression in Cy3 if possible.

Note

Check to make sure the dyes are different when making cocktails with DCs. For P/S antibodies, different species as well as different dyes must be used for each antibody. For example, a mouse IgG1 antibody and a rabbit IgG antibody could be pooled; an example of pooled secondary antibodies to use would Cy3-Donkey-anti-mouse and Cy5-Donkey-anti-rabbit.

Ab Characterization

14 If you are trying to characterize a new primary-secondary antibody, refer to **Study Design & Process Flow** (Step 27)

15 If you are trying to validate a new directly conjugated antibody, proceed to **New Direct Conjugate Validation Study Design** (Step 72).

16 If you are trying to re-validate a directly conjugated antibody (new lot/batch), proceed to **Revalidating Directly Conjugated Antibodies** (Step 86).

17 If you are trying to validate Zenon labelling, proceed to **Zenon Labeling** (step 98).

18 If you are studying antigen effects for a given antibody, proceed to **Overview of Experimental Design for Testing Antigen Effects to Dye Inactivation** (step 123).

Coverslipping

19 Slides are coverslipped to protect the tissue from drying out.

20 Mounting media should be removed from the 4 °C fridge and brought to Room temperature .

21 Slides should be quickly dipped in ddH2O.

22 Take a rainin pipet tip (200µL-green box) and with a scissors clip the end to make a wide bore.

23 Take up 75 µL mounting media (4% propyl gallate, 1% DABCO, 90% glycerol) and add to one end of the slide. For leaching markers, use alternate mounting media (50% glycerol, 4% propyl gallate).

24 Place the end of the coverslip at a 45 degree angle and slowly lower; allowing the mounting media to flow under the coverslip across the slide.

25 Make sure there is not mounting media oozing out of the sides of the coverslip; remove any excess by gently blotting on a Kimwipe.

26 Proceed to Imaging as per the Cell DIVE™ Imaging manual or store the slides at 4 °C in light protected environment. (Recommendation: image within 24 hours of staining.)

Study Design & Process Flow

27 Below is a schematic that provides an overview of the experiment design for antibody characterization for primary-secondary staining. Depending on the slide type (ie, cell pellet vs TMA) and scenario (ie, controls on the slide), a certain experimental design is required. The user should map their scenario to the corresponding study design they must execute.

A	B	C	D	E	F
Scenario		Slide 1	Slide 2	Slide 3	Slide 4
	Cell pellet with an absolute positive & negative control	Stain with primary/secondary antibodies			

A	B	C	D	E	F
Cell Pellets	Cell pellet with or without an absolute positive & negative control; biomarker is a phospho target	Stain with primary/secondary antibodies	Phosphatase treatment, followed by primary/ secondary stain		
	Cell pellet with or without an absolute positive & negative control; primary antibody has a peptide block	Stain with primary/secondary antibodies	Peptide block with primary/secondary stain		
	Cell pellet with or without an absolute positive & negative control; primary antibody does not have a peptide block	Stain with primary/secondary antibodies	Stain with isotype control followed by secondary stain		
TMA	TMA with positive and negative control tissue and biomarker is a phospho target	Stain with primary/secondary antibodies	Phosphatase treatment, followed by primary/secondary stain		
	TMA with positive and negative control tissue; primary antibody has a peptide block	Stain with primary/secondary antibodies	Peptide block with primary/secondary stain		
	TMA with positive and negative control tissue; primary antibody does not have peptide block	Stain with primary/secondary antibodies	Stain with isotype control followed by secondary stain		
	TMA does not exist with both positive and negative control tissue; biomarker is a phospho target	Positive control slide: Stain with primary/secondary antibodies	Positive control slide: Phosphatase treatment, followed by primary/secondary stain	Negative control slide: Stain with primary/secondary antibodies	Negative control slide: Phosphatase treatment, followed by primary/secondary stain

A	B	C	D	E	F
	TMA does not exist with both positive and negative control tissue; primary antibody has a peptide block	Positive control slide: Stain with primary/secondary antibodies	Positive control slide: Peptide block with primary/secondary stain	Negative control slide: Stain with primary/secondary antibodies	Negative control slide: Peptide block with primary/secondary stain
	TMA does not exist with both positive and negative control tissue; primary antibody has no peptide block	Positive control slide: Stain with primary/secondary antibodies	Positive control slide: Stain with isotype control followed by secondary stain	Negative control slide: Stain with primary/secondary antibodies	Negative control slide: Stain with isotype control followed by secondary stain

Overview of the experiment design for antibody characterization for primary-secondary staining.

28 Once the study design has been selected, the user must follow the corresponding process flow for the design. The table below captures the required process for each study design.

A	B	C	D
Primary/Secondary Antibody Stain	Primary/Secondary Antibody Stain with Phosphatase Pretreatment	Primary/Secondary Antibody Stain with Immunizing Peptide Block	Primary/Secondary Antibody Stain with Isotype Controls
Clear Slides	Clear Slides	Clear Slides	Clear Slides
Antigen Retrieval	Antigen Retrieval	Antigen Retrieval	Antigen Retrieval
	Phosphatase Pretreatment		
Block Slides	Block Slides	Block Slides	Block Slides
Dapi Stain, coverslip, background image	Dapi Stain, coverslip, background image	Dapi Stain, coverslip, background image	Dapi Stain, coverslip, background image
		Perform peptide & Primary Antibody Incubation	
Stain Slides	Stain Slides	Stain Slides	Stain slides with isotype control alongside
Image	Image	Image	Image
Qualitative Assessment	Qualitative Assessment	Qualitative Assessment	Qualitative Assessment
Quantitative Assessment	Quantitative Assessment	Quantitative Assessment	Quantitative Assessment

Process Based on Experimental Characterization Scheme

29 If your study requires Phosphatase Pretreatment, proceed to **Controls—Phosphatase Pretreatment of Tissue** (step 51).

30 If your study requires Immunizing Peptide Block, proceed to **Controls—Blocking with Immunizing Peptide** (step 60).

31 If your study requires Isotype Controls, proceed to **Controls—Isotype Controls** (step 66).

Primary-Secondary Staining

2h 15m

32 Retrieve primary antibody from the 4 °C walk-in refrigerator or -20 °C freezer. Be sure to keep On ice **at all times**.

33 Spin the stock antibody down at 13000 x g, 00:01:00 . If stock antibodies are in large or odd shaped vials, transfer them to eppendorf tubes and label appropriately with a LIMS sticker (if possible, remove the LIMS label from the original vial and stick it on the eppendorf tube).

34 Make up primary antibody dilutions in the antibody diluent solution ([M] 3 % (v/v) BSA / 1 x PBS) using the manufacturer suggested concentration of antibody, with the exceptions noted.

Note

- If there is no concentration suggested, then use 5 µg/ mL.
- If the vendor suggests < 1 µg/ mL, then increase it to 1µg/ mL (when performing 01:00:00 Room temperature incubation).
- If no concentration or recommended dilution is available, use a 1:100 dilution.

35 Spin down dilutions at 13000 x g, 00:01:00 and keep dilutions On ice . Put antibodies back to proper storage location after making up dilutions.

36 Slides should be in 1 x PBS (from decoverslipping step); remove from PBS and turn slide on its side and gently tap slide on blue pad/Kimwipe to remove solution. Remove as much of the PBS solution without letting the tissue dry out and without touching the tissue.

37 Place slides in humidified chamber. Chamber should contain water in the bottom. Move quickly in adding the primary antibody so that slides do not dry out.

38 Add a total of 200 µL antibody dilution to the slide; cover with parafilm cut to a size just larger than tissue area. Make sure there are no bubbles under the parafilm. Place lid on humidified chamber.

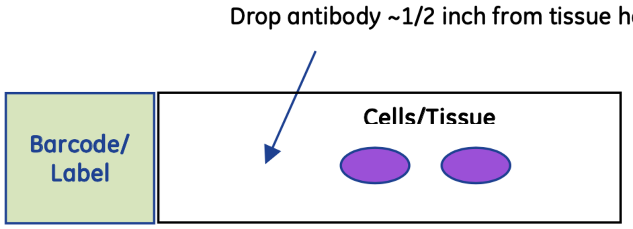

39 Incubate for 01:00:00 at Room temperature . 1h

40 Retrieve secondary antibody from -20 °C ; keep On ice and then spin down at 13000 x g, 00:01:00 .

41 Make up secondary antibody dilutions in the antibody diluent solution ([M] 3 % (v/v) BSA / 1 x PBS). Secondary antibodies are stained at a final concentration of [M] 5 microgram per milliliter (µg/mL) .

42 Spin down dilutions for 13000 x g, 00:01:00 . Keep On ice in light protected environment while the primary antibody incubation is taking place.

43 After primary antibody incubation is complete, remove parafilm and place slides in coplin jar or staining dish.

44 Wash slides for 3x 00:05:00 with 1 x PBS , using gentle agitation on shaker. 15m

45 After final PBS wash, turn slide on its side and gently tap slide on blue pad/Kimwipe to remove remaining PBS. Once again remove as much of the liquid without letting the tissue dry out and without touching the tissue.

46 Place slides in humidified chamber. Add a total of 200 µL secondary antibody dilution to the slide just as suggested above. Cover with parafilm cut to a size just larger than tissue area. Make sure there are no bubbles under the parafilm. Place lid on humidified chamber.

47 Incubate for 01:00:00 at Room temperature . 1h

48 Remove parafilm and place slides in coplin jar or staining dish.

49 Wash slides for 3x 00:05:00 with 1 x PBS , using gentle agitation on shaker. 15m

50 Proceed *immediately* to **Coverslipping**. go to step #19

Controls—Phosphatase Pretreatment of Tissue

2h 25m

51

Note

The phosphatase pretreatment of tissue must occur prior to the slide blocking that occurs in Cell DIVE™ Platform | Slide Clearing and Antigen Retrieval. The user is responsible for following the appropriate process flow given the study design for the antibody characterization.

52 Slides should be coming out of 1 x PBS from antigen retrieval.

53 Rinse slides for 3x 00:05:00 with **ddH_2O**. 15m

54 Check to make sure the oven is at 37 °C .

55 Locate the Lambda Protein Phosphatase - 20,000 units **New England Biolabs Catalog #P0753S** which should be stored in -80 °C freezer.

Keep the phosphatase On ice at all times.
The 10X buffer and $MnCl_2$ may be thawed and then kept On ice .

56 Make up the following solutions:

A	B	C
Kit Reagents	No phosphatase pretreament	Phosphatase pretreament
10x buffer	20 uL	20 uL
10x MnCl2	20 uL	20 uL
Lambda	0 uL	8 uL
ddH20	160 uL	152 uL
Total Volume	200 uL	200 uL

57 Add entire volume (200 µL) to slides and incubate at 37 °C for 02:00:00 in humidified chamber. 2h
Do not cover slides with parafilm.

58 Wash slides for 2x 00:05:00 each in ddH_2O with gentle agitation. 10m

59 Proceed to Slide Blocking in Cell DIVE™ Platform | Slide Clearing and Antigen Retrieval for the blocking procedure.

Controls—Blocking with Immunizing Peptide

30m

60 The peptide blocking experiment should be done alongside a primary/secondary antibody staining experiment.

61 Retrieve peptide from 4 °C walk-in refrigerator or -20 °C freezer and keep On ice .

62 Use the manufacturer's suggested concentration of peptide (if not indicated on datasheet call vendor); if there is not a concentration recommended then use 100-fold molar excess of peptide to antibody.

Note

If you are unsure in how to calculate this please consult your study coordinator.

63 Use the concentration of primary antibody suggested as per go to step #34 in the **Primary - Secondary Antibody** Section.

64 Dilute peptide and primary antibody in the antibody diluent buffer ([M] 3 % (v/v) BSA / 1 x PBS); mix thoroughly and follow vendor's recommended incubation period. If there is not one suggested then allow reaction to incubate for 00:30:00 at Room temperature . 30m

65 Add peptide & primary antibody solution to slide and follow remaining steps in **Primary - Secondary Antibody** starting at go to step #36 .

Controls—Isotype Controls

66 The isotype control experiment should be done alongside a primary/secondary antibody staining experiment. The isotype control employed should match the IgG type of the primary antibody.

Note

The same isotype control can be used for antibodies that have the same IgG type if the final concentration used for the antibodies is ≤ 5µg/mL. Any antibody that has a final concentration of > 5µg/mL needs its own isotype control even though it may have the same IgG type as another antibody.

67 Double-check the correct isotype control is being used.

68 If the isotype is a Mouse IgG1 or Rabbit IgG1, use the Cell Signaling Isotype controls. If it is Mouse IgG2a,2b or Rat, etc use the R&D isotype controls.

69 Retrieve isotype control from 4 °C or -20 °C storage location and keep On ice .

70 Use equivalent concentration of isotype control as what is used for the primary antibody. Dilute the isotype control in the antibody diluent buffer ([M] 3 % (v/v) BSA / 1 x PBS).

71 Proceed to **Primary - Secondary Antibody** starting at go to step #36 .

New Direct Conjugate Validation Study Design

72 Refer to the below table to review the experimental design for validating directly conjugated antibodies.

A	B	C	D	E	F	G
Treatment						
Primary/Secondary Antibody staining	Slide 1					
Directly Conjugated Antibody (D:P ratio1), 5 ug/mL		Slide 2				
Directly Conjugated Antibody (D:P ratio1), 10ug/mL			Slide 3			
Directly Conjugated Antibody (D:P ratio2), 5 ug/mL				Slide 4		

	A	B	C	D	E	F	G
	Directly Conjugated Antibody (D:P ratio2), 10ug/mL					Slide 5	

Overview of Experimental Design for Validating Directly Conjugated Antibodies

73 The slides used for this experiment should be the same type that was used in phase I of the **Primary-Secondary Staining**.

74 For the Primary-Secondary Staining slide, use the purified form of the primary antibody if the antibody was purified prior to the conjugation (test at the same concentration used in the P/S experiment).

Staining with Directly Conjugated Antibodies

1h 15m

75 Retrieve primary antibody and direct conjugates from the 4 °C walk-in refrigerator or -20 °C freezer. Be sure to keep On ice at all times.

76 Spin the stock antibody down at 13000 x g, 00:01:00 .

77 Make up necessary primary antibody & direct conjugate antibody dilutions in the antibody diluent buffer ([M] 3 % (v/v) BSA / 1 x PBS) using amber tubes for direct conjugates.

Note

The concentration for the unconjugated primary antibody should be equivalent to what was used in the initial phase I characterization experiment. For the direct conjugates use [M] 5 microgram per milliliter (µg/mL) & [M] 10 microgram per milliliter (µg/mL) .

Note

In the event that the primary antibody was used at [M] 20 microgram per milliliter (µg/mL) for the initial characterization; then the direct conjugate should be stained with [M] 20 microgram per milliliter (µg/mL) and [M] 30 microgram per milliliter (µg/mL) of the direct conjugate for an extended period of time, 02:30:00 .

78 Spin the dilutions at 13000 rpm, 00:01:00 and keep dilutions On ice . Put antibodies and direct conjugates back to proper storage location after making up dilutions.

79 Slides should be in 1 x PBS (from decoverslipping step); remove from PBS and turn slide on its side and gently tap slide on blue pad/Kimwipe to remove solution. Remove as much of the PBS solution without letting the tissue dry out and without touching the tissue.

80 Place slides in humidified chamber. Chamber should contain water in the bottom. Move quickly in adding the primary antibody so that slides do not dry out.

81 Add a total of 200 µL antibody dilution to the slide; cover with parafilm cut to a size just larger than tissue area. Make sure there are no bubbles under the parafilm. Place lid on humidified chamber.

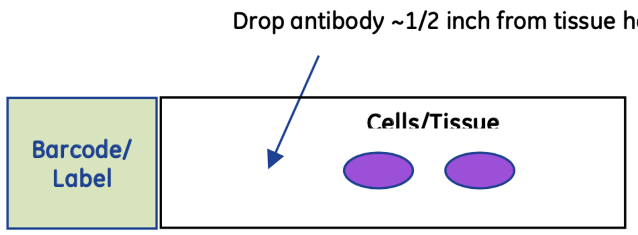

82 Incubate for 01:00:00 at Room temperature . 1h

83 Remove parafilm and place slides in coplin jar or staining dish.

84 Wash slides 3x 00:05:00 with 1 x PBS, using gentle agitation on shaker. 15m

85 Proceed *immediately* to **Coverslipping**. go to step #19

Revalidating Directly Conjugated Antibodies

86 Refer to the below table to review the experimental design for re-validating directly conjugated antibodies.

- A total of four slides are required.
- 1 slide is stained with the old lot, and the remaining 3 are stained with different concentrations of the new lot.

A	B	C	D	E
Treatment				
Old Lot of direct conjugate	Slide 1			
New Lot of direct conjugate (5 ug/mL)		Slide 2		
New Lot of direct conjugate (10 ug/mL)			Slide 3	
New Lot of direct conjugate (15 ug/mL)				Slide 4

Overview of Experimental Design for Re-Validating Directly Conjugated Antibodies

Note

The slides used for this experiment must be tissue representative of what will be employed for the multiplexing experiment. The purpose is to determine the optimal concentration of direct conjugate to employ for multiplexing.

Staining with Directly Conjugated Antibodies

87 Retrieve direct conjugates from the 4 °C refrigerator. **Be sure to keep** On ice **at all times.**

88 Spin the direct conjugates down at 13000 x g, 00:01:00 .

89 Make up necessary direct conjugate dilutions in the antibody diluent buffer ([M] 3 % (v/v) BSA / 1 x PBS) using amber tubes.

The new direct conjugate concentrations should have a range with the working concentration of the old direct conjugate as the mid-point.

Note

For example if the old DC had a working concentration of [M] 10 microgram per milliliter (µg/mL) then the new DC should be tested at concentrations of [M] 5 microgram per milliliter (µg/mL) , [M] 10 microgram per milliliter (µg/mL) and [M] 15 microgram per milliliter (µg/mL) .

90 Spin the dilutions at 13000 rpm, 00:01:00 and keep dilutions On ice . Put direct conjugates back to proper storage location after making up dilutions.

91 Slides should be in 1 x PBS (from decoverslipping step); remove from PBS and turn slide on its side and gently tap slide on blue pad/Kimwipe to remove solution. Remove as much of the PBS solution without letting the tissue dry out and without touching the tissue.

92 Place slides in humidified chamber. Chamber should contain water in the bottom. Move quickly in adding the DC so that slides do not dry out.

93 Add a total of 200 µL antibody dilution to the slide; cover with parafilm cut to a size just larger than tissue area. Make sure there are no bubbles under the parafilm. Place lid on humidified chamber.

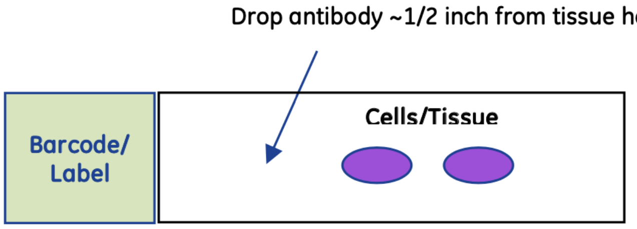

94 Incubate for 01:00:00 at Room temperature . 1h

95 Remove parafilm and place slides in coplin jar or staining dish.

96 Wash slides 3x for 00:05:00 with 1 x PBS , using gentle agitation on shaker. 15m

97 Proceed *immediately* to **Coverslipping**. go to step #19

Zenon Labeling Antibodies

20m

98 Zenon labeling provides a fast alternative way of conjugating antibodies. An unlabeled antibody is incubated with fluorophore-labeled Fab fragment and then mixed with a non-specific IgG to neutralize any extra unbound Fab fragments. The initial antibody does not require purification, prior to or after labeling.

An initial zenon experiment begins by targettng a 3:1 dye to protein ratio and staining at concentrations of [M] 10 microgram per milliliter (µg/mL) and [M] 20 microgram per milliliter (µg/mL) .

A	B
Zenon Kit	Vendor and Cat. No.
Zenon® Alexa Fluor® 555 Rabbit IgG	Invitrogen Z25305
Zenon® Alexa Fluor® 555 Mouse IgG1	Invitrogen Z25005
Zenon® Alexa Fluor® 555 Mouse IgG2b	Invitrogen Z25205
Zenon® Alexa Fluor® 647 Rabbit IgG	Invitrogen Z25308
Zenon® Alexa Fluor® 647 Mouse IgG1	Invitrogen Z25008
Zenon® Alexa Fluor® 647 Mouse IgG2b	Invitrogen Z25208

Zenon labeling kits from Invitrogen

Note

Use amber tubes during the workflow.

Validating zenon labeling

20m

99 Zenon labeling validation is a 3 slide experiment: slide 1 - P/S, slides 2 and 3 - titration of zenon complexes. Prepare 3 bullet tubes for the initial zenon labeling validation.

100 The IgG concentration of Ab should be known. If unknown, contact vendor. If no concentration can be supplied, assume 200 µg/mL.

101 Initial zenon labeling should be done using 2 µg and 4 µg antibody with a relevant IgG (rabbit IgG or mouse IgG1, etc.) labeling reagent when using a final staining volume of 200 µL.

This results in a final concentrations of [M] 10 microgram per milliliter (µg/mL) and [M] 20 microgram per milliliter (µg/mL), respectively.

102 Volume of Ab needed for each initial validation should not be more than 20 µL.

103 For each concentration, test a 3:1 molar ratio of zenon to antibody target to begin.

103.1 Using 2 µg antibody at 3:1 ratio (slide 2 of the validation):

- Take 2 µg antibody and add 10 µL labeling reagent. (Pipet up and down to mix instead of vortexing due to low volumes).

103.2 Using 4 µg antibody at 3:1 ratio (slide 3 of the validation):

- Take 4 µg antibody and add 20 µL labeling reagent. (Pipet up and down to mix instead of vortexing due to low volumes).

104 Incubate both tubes for at least 00:15:00 at Room temperature . 15m

105 Add an amount of zenon blocking reagent to each tube equivalent to the amount of labeling reagent used (i.e. 10 µL for Tube 2, 20 µL for Tube 3).

106 Incubate for 00:05:00 at Room temperature . 5m

107 Bring the volume of each tube to 200 µL using antibody diluent ([M] 3 % (v/v) BSA / 1 x PBS) for the manual staining.

108 For Tube 1, prepare the P/S antibody diluted to 200 µL at the appropriate staining concentration. (Staining concentration as per vendor recommendation or based on previous staining data.)

> **Note**
>
> Conjugates must be applied to slides within 00:30:00 .

109 Slides should be in 1 x PBS (from decoverslipping step); remove from PBS and turn slide on its side and gently tap slide on blue pad/Kimwipe to remove solution. Remove as much of the PBS solution without letting the tissue dry out and without touching the tissue.

110 Place slides in humidified chamber. Chamber should contain water in the bottom. Move quickly in adding the primary antibody so that slides do not dry out.

111 Add a total of 200 µL antibody dilution to each slide; cover with parafilm cut to a size just larger than tissue area. Make sure there are no bubbles under the parafilm. Place lid on humidified chamber.

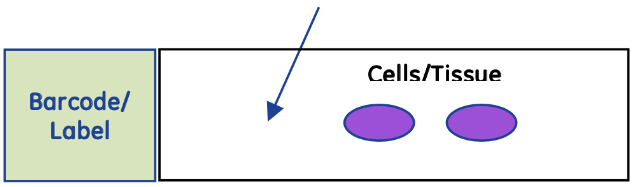

112 Incubate for 01:00:00 at Room temperature .

113 Remove parafilm and place slides in coplin jar or staining dish.

114 Wash slides 3x for 00:05:00 with 1 x PBS , using gentle agitation on shaker. 15m

115 Proceed *immediately* to **Coverslipping**. go to step #19

If initial Zenon validation didn't work

1h 50m

116 If the initial zenon validation did not work, consider testing a lower ratio of zenon to antibody target. Target 1.5 -- half the dye to protein targeted initially -- at final concentrations of [M] 10 microgram per milliliter (µg/mL) and [M] 20 microgram per milliliter (µg/mL) .

116.1 Using 2 µg antibody when using a final staining volume of 200 µL :

- Take 2 µg antibody and add 2 µL labeling reagent . (Pipet up and down to mix instead of vortexing due to low volumes).

116.2 Using 4 µg antibody when using a final staining volume of 200 µL :

- Take 4 µg antibody and add 4 µL labeling reagent . (Pipet up and down to mix instead of vortexing due to low volumes).

117 Incubate both tubes for at least 00:15:00 at Room temperature . 15m

118 Add an amount of zenon blocking reagent to each tube equivalent to the amount of labeling reagent used. (i.e. 2 µL for Tube 2, 4 µL for Tube 3)

119 Incubate for 00:05:00 at Room temperature . 5m

120 Bring the volume of each tube to 200 µL using antibody diluent ([M] 3 % (v/v) BSA / 1 x PBS) for the manual staining.

121 Conjugates must be applied to slides within 00:30:00 . 30m

122 Stain for 01:00:00 at Room temperature . 1h

Overview of Experimental Design for Testing Antigen Effects to Dye... 15m

123 There should be a total of 4 slides prepared for testing the dye inactivation/bleaching effects. Slide 1 **will not** be treated with the dye inactivation solution, Slide 2 will be treated with 1 round of dye inactivation, Slide 3 will be treated with 5 rounds of dye inactivation, and Slide 4 will be treated with 10 rounds of dye inactivation.

A	B	C	D	E
Treatment				
No Dye Inactivation	Slide 1			
1 Round of Dye Inactivation		Slide 2		
5 Rounds of Dye Inactivation			Slide 3	
10 Rounds of Dye Inactivation				Slide 4

Experimental slides used for determining antigen effects for an antibody.

124 Slides should be coming out of 1 x PBS from the antigen retrieval.

125 Keep slide 1 in 1 x PBS.

126 Be sure that the 0.5 M $NaHCO_3$ has had a pH read within 7 days.

127 Make fresh dye inactivation solution.

Note

Refer to the working solution for dye inactivation. The hydrogen peroxide is added to the solution immediately before adding to the staining dish. This reaction is time sensitive.

128 Within one minute of making the dye inactivation solution, pipet 500 µL onto slides 2, 3, and 4 in a humidified chamber containing water. (If there are many slides, use a staining dish containing 200 mL solution).

129 Incubate at Room temperature for 00:15:00. Do not place parafilm over the slides. 15m

130 Remove slide 2 and transfer to 1 x PBS. Wash 3x for 5 minutes with gentle agitation on shaker and hold in PBS.

131 Perform a quick 1 x PBS wash (less than one minute) for slides 3 and 4 and continue bleaching, making a fresh dye inactivation solution for each round.

132 For slide 3, continue to 5 rounds of dye inactivation and for slide 4 continue to 10 rounds of dye inactivation. Perform a quick 1 x PBS wash between each round.

133 After the final round, wash slides 3x for 00:05:00 with 1 x PBS, using gentle agitation on shaker. 15m

134 Proceed *immediately* to **Coverslipping**. go to step #19

135 Acquire background images for the slides as per the Cell DIVE Imaging Manual.

136 Decoverslip the slides and stain all 4 slides with the same antibody, as per **Primary-Secondary Staining** or **DC staining**, depending on the Ab being tested for dye inactivation.

- Use the validated concentration from the pervious experiments.

137 Proceed *immediately* to **Coverslipping**. go to step #19

138 Acquire background images for the slides as per the Cell DIVE Imaging Manual.

Go/No Go Decision: Primary Secondary Antibody staining

139 Open images of all slides in your viewer of choice (FIJI or QUPath, for example).

140 Compare antibody images to corresponding control slides.

- Refer to Table 1: Image Analysis and Comparison

	A	B	C
	Type Of Comparison	Type Of Evaluation	Normalized To
	Specificity	• primary/secondary vs. Isotype controls or phosphatase pretreatment<br>• three positives and one negative control tissue<br>• if there is not a negative control use an internal negative control	primary/secondary
	Specificity	• Primary/secondary of antibody 1 vs. Primary/secondary of antibody 2 vs. primary/secondary of antibody 3 vs. DAB	Auto-level each
	Difference in intensity	• Primary/secondary of antibody 1 vs. Primary/secondary of antibody 2 vs. primary/secondary of antibody 3	To brightest specific primary

Table 1: Image Analysis and Comparison

141 Compare antibody staining from all vendors to each other.

142 Compare antibody staining to DAB images, if available.

143 There should be a maximal signal to noise ratio.

144 Select the antibody with the best specificity (localization same as vendor/literature/collaborator suggested) and signal.

145 For troubleshooting on staining, refer to Figure 1: Troubleshooting Primary Antibody Characterization Flow Chart.

Note

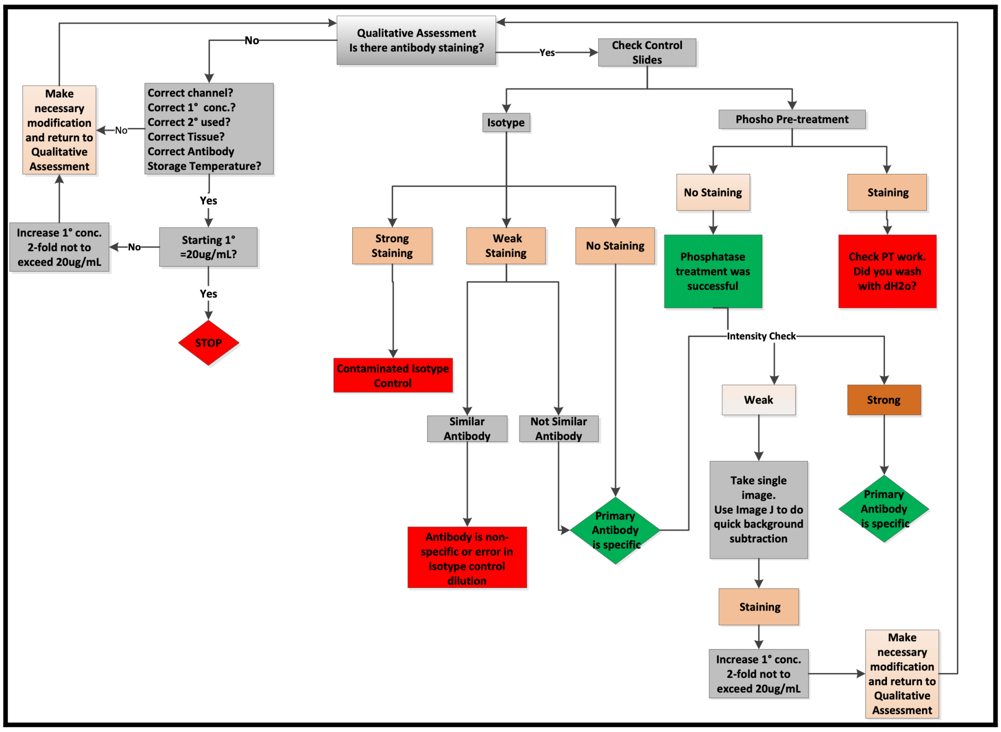

Figure 1: Troubleshooting Primary Antibody Characterization Flow Chart

146 Note: If performing Primary/Secondary staining in the first round of staining of a multiplex, an IgG block to the isotype of the primary antibodies used, should be performed at a 10-fold concentration. For example: If isotypes of the primary antibodies were mouse IgG and Rabbit IgG, then after bleaching the first round of staining, slides should be blocked in a cocktail rabbit and mouse serum for 30 minutes before proceeding to the second round of staining.

Go/No Go Decision: Direct Conjugates

147 The direct conjugate staining should have comparable localization to the primary/secondary antibody and DAB images.

- Refer to Table 2: Image Analysis And Comparison

A	B	C
Type Of Comparison	Type Of Evaluation	Normalized To
Specificity	primary/secondary vs. direct conjugates	Auto-level each
Intensity	primary/secondary vs. direct conjugates	Normalize to primary/secondary
Intensity	direct conjugates vs. direct conjugates	Normalize to brightest most specific direct conjugate

Table 2: Image Analysis and Comparison

148 Down select the D/P and concentration that yields the maximal signal to noise ratio.

149 For the troubleshooting workflow, refer to Figure 2: Direct Conjugate Troubleshooting Workflow.

Note

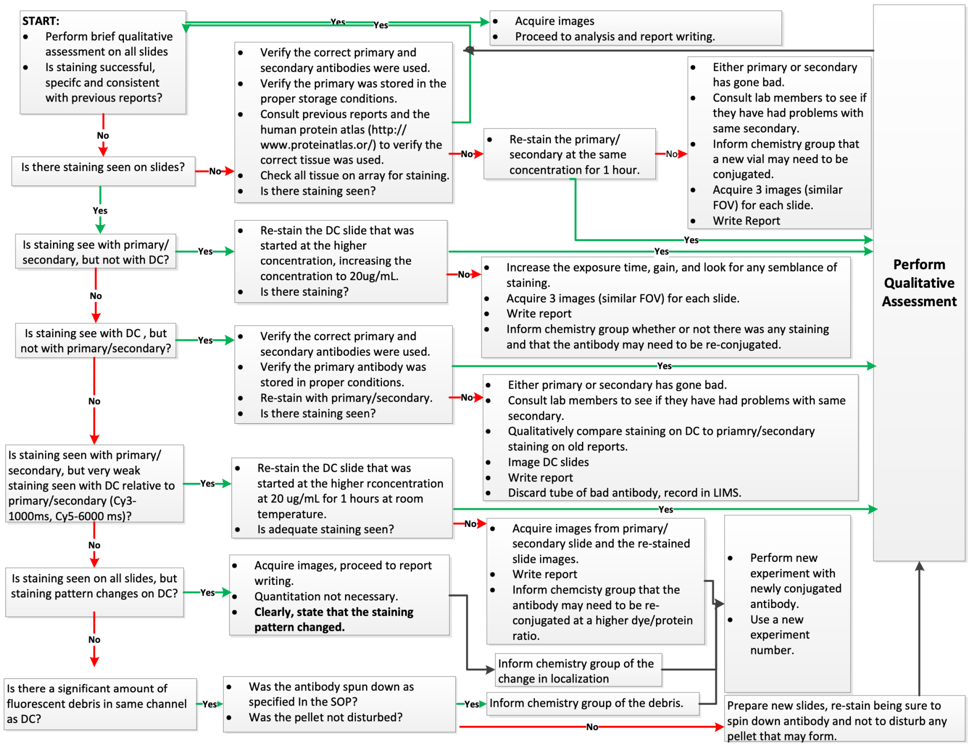

Figure 2: Direct Conjugate Troubleshooting Workflow

150 Note: If an antibody is not successful as a direct conjugate, options for including that antibody in a multiplex are as follows:

- Try validation with another dye
- Use with secondary detection. If performing Primary/Secondary staining, an IgG block to the isotype of the primary antibodies used should be performed at a 10-fold concentration. This should occur before staining a direct conjugate raised in the same species. For example: If isotypes of the primary antibodies were mouse IgG and Rabbit IgG, then after dye inactivating that round of staining, slides should be blocked in a cocktail rabbit and mouse serum for 30 minutes before proceeding to the next round of staining.
- Look for a commercial conjugate

- Try zenon labeling

Go/No Go Decision: Revalidating Direct Conjugates

151 The new direct conjugate staining should have comparable localization to the old direct conjugate lot.

- Refer to Table 3: Image Analysis And Comparison

A	B	C
To Be Compared	To Be Evaluated	Normalized To
Specificity	OLD direct conjugate vs. NEW direct conjugate vs. primary/secondary antibody vs. DAB	Auto-level each
Intensity	OLD direct conjugate vs. NEW direct conjugate	Normalize to old lot

Table 3: Image Analysis And Comparison

152 Maximize the signal to noise ratio.

Determining Antigen Effects

153 Select the treated slide with the "no dye inactivation" slide (Slide 1).

- Refer to Table 4 : Dye Inactivation Slide Comparison And Evaluation.

A	B	C
Type Of Comparison	Type Of Evaluation	Normalized To
Specificity	"No Dye Inactivation" vs. historical images from primary down selection experiment vs. DAB images	Auto-level each
Specificity	"No Dye Inactivation" vs. dye inactivation round	Auto-level each
Difference in intensity	"No Dye Inactivation" vs. dye inactivation round	"No Dye Inactivation" slide

Table 4: Dye Inactivation Slide Comparison And Evaluation

154 No antigen effects from dye inactivation is defined as:

- No greater than 20% loss in stain intensity between "no dye inactivation" vs. other rounds.
- Stain specificity is maintained.
- Tissue integrity is maintained.
- Non specific background staining is minimal.

Note

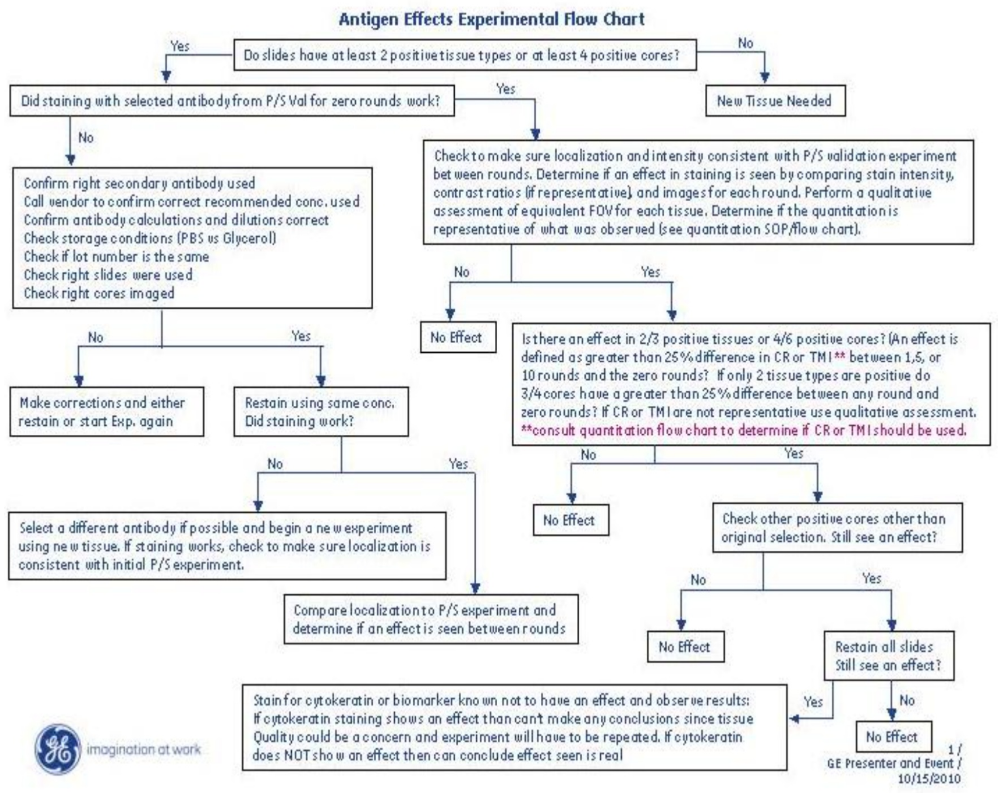

Figure 3: Antigen Effects Troubleshooting Workflow

155 There should be a maximal signal to noise ratio.
