# Supplementary material for: Pathobiology of Candida auris infection analyzed by multiplexed imaging and single cell analysis
Source: PLoS One. 2024 Jan 17;19(1):e0293011. doi: 10.1371/journal.pone.0293011 (PMC10793899; doi:10.1371/journal.pone.0293011)
Supplement: S2 File — Protocol document from protocol io (dx.doi.org/10.17504/protocols.io.bpv6mn9e), which outlines antibody staining and imaging for multiplexing. (PDF) [file pone.0293011.s002.pdf]

Cell DIVE™ Platform | Antibody Staining & Imaging

Liz McDonough[1], Chrystal Chadwick[1], Fiona Ginty[1], Christine Surrette[1], Anup Sood[1]

[1]GE Research

Human BioMolecular Atlas Program (HuBMAP) Method Development Community | GE F

Liz McDonough
GE Research

DEC 15, 2020

OPEN ACCESS

DOI: dx.doi.org/10.17504/protocols.io.bpv6mn9e

Protocol Citation: Liz McDonough, Chrystal Chadwick, Fiona Ginty, Christine Surrette, Anup Sood 2020. Cell DIVE™ Platform | Antibody Staining & Imaging. **protocols.io** https://dx.doi.org/10.17504/protocols.io.bpv6mn9e

License: This is an open access protocol distributed under the terms of the Creative Commons Attribution License, which permits unrestricted use, distribution, and reproduction in any medium, provided the original author and source are credited

Protocol status: Working
We use this protocol and it's working

Created: Nov 19, 2020

Last Modified: Dec 15, 2020

PROTOCOL integer ID: 44702

Keywords: Cell DIVE™, FFPE, staining, multiplexed immunofluorescence

ABSTRACT

This document outlines the workflow for antibody staining, dye inactivation, and imaging as per the Cell DIVE™ technology.

ATTACHMENTS

Cell_DIVE-Ab-staining-and-imaging_final-version.pdf

GUIDELINES

Troubleshooting

Primary Secondary Antibody Staining

Figure 1: Troubleshooting Primary Antibody Characterization Flow Chart

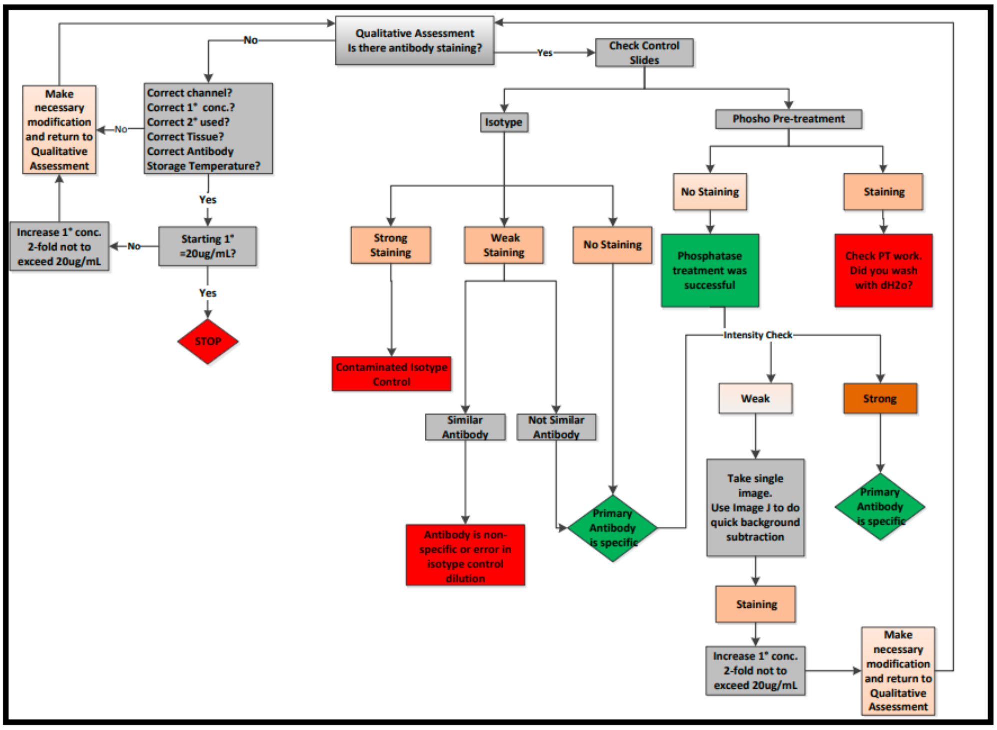

Direct Conjugates

Figure 2: Direct Conjugate Troubleshooting Workflow

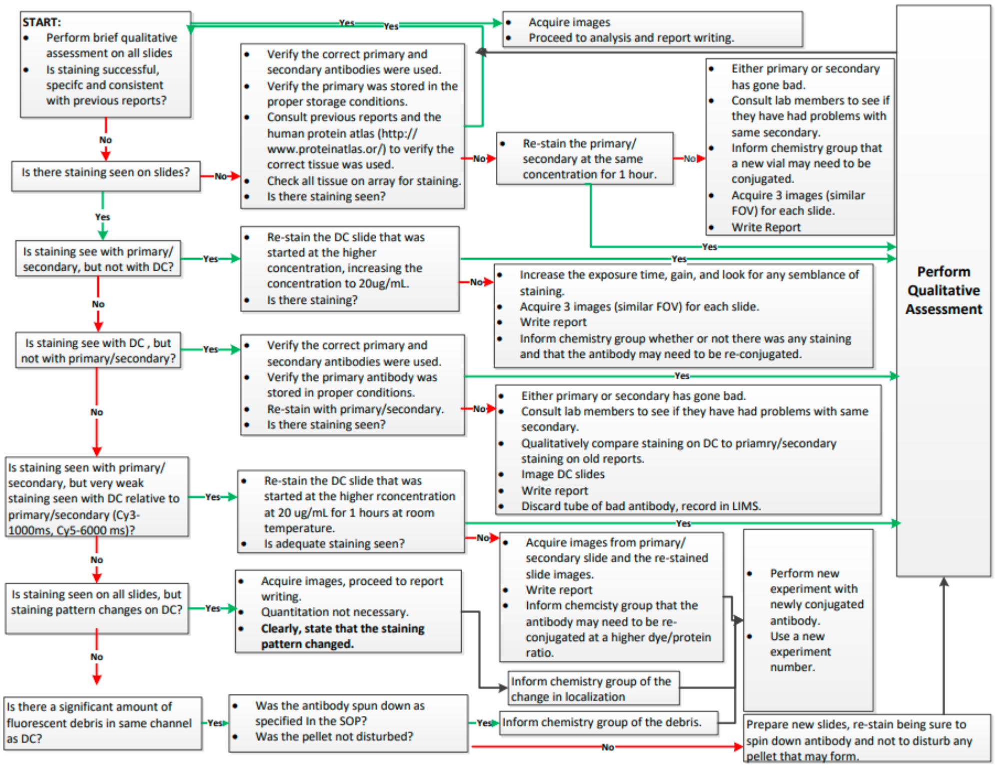

MATERIALS

Required Materials

Table 2: Summary of the required materials for the workflow.

A	B
Material	Definitions
0.45µm filter	Filtration of mounting media
Amber glass bottles	Storage for mounting media and/or DAPI
Amber 1.5mL Eppendorf tubes	Storage for antibody direct conjugates
50mL conical tubes	Storage for Antibody diluent
Weigh Boats	Used to weigh out solid/powder reagents on analytical balance
Coverslips	Used to coverslip the tissue slides
Pipette Tips (0.5-1000µL)	Used for pipettes
Serological Pipettes	Deliver liquid volumes (mL) for solutions
Slide Boxes/Holders	Holders or Boxes for slide storage and different incubations (ie, coplin jars)
Transfer Pipettes	Delivers PBS for decoverslipping
Lab Tape	Labels bottles and tally scoring

A	B
Xylene-Resistant slide labels	Labels which are resistant to xylene
Nitrile Gloves	Personal Protective Equipment
Kimwipes (large and small)	Clean mounting media from slides
Blue Underpads	Underpad used to absorb reagent spills on the work bench

Required Reagents and Stock Solutions

Table 3: Summary of the required stock solutions and reagents for the workflow

A	B
Stock Solutions	Expiration
DAPI Stock Solution	10 mg vial of DAPI dilactate<br>2.0 mL ddH20<br>----------------------<br>2 mL Total Volume<br><br>Mix thoroughly until solid is dissolved.<br>Aliquot into (20) 100 uL aliquots into amber eppendorfs.<br>Store in -20 deg C
Jackson Immuno Cy-Dye Secondary Antibodies	Secondary antibodies should be reconstituted in ddH20 to a final<br>concentration of 1mg/mL. Make 5ul aliquot in amber tubes and<br>store at -20 deg C up to 6 months.
0.5 M NaHCO3 , pH 11.2<br>(acceptable range 10.9-11.3)	42 g NaHCO3<br>1000 mL ddH20<br>----------------------<br>1000 mL Total Volume<br><br>Mix thoroughly until solid is dissolved thoroughly and pH ~11.2<br>(~15-18g of NaOH pellets).<br><br>The acceptable range for pH is between 10.9 and 11.3. Check and log the pH on a weekly basis during periods of use. If the pH is measured outside of this specification, discard the buffer and do not use.<br>Store at 4C for up to 6 months.

The below table contains the respective expiration and storage condition for each stock reagent or solution.

Table 4: Summary of the required reagents and stock solutions, and their respective shelf time and storage conditions.

A	B	C
Solutions	Expiration	Storage
Liquid Chemicals		
Ethanol	2 years from receipt	RT-flammable cabinet
DABCO 4mM	1 week	4 degrees C

A	B	C
Phosphate buffered saline	1 year from receipt	RT
DAPI	1 year from receipt	-20
Glycerol	--	RT
30% Hydrogen Peroxide	6 months from receipt	4 degrees C
Dry Chemicals		
Propyl gallate	2 years from receipt	RT
DABCO	2 years from receipt	4 degrees C
BSA	2 years from receipt	4 degrees C
Donkey Serum		-20 degrees C
Sodium bicarbonate	2 years from receipt	RT
Sodium Hydroxide Pellets	2 years from receipt	RT

Validated Antibodies

Refer to the GE primary antibody catalog and the directly conjugate (DC) antibody catalog for the list of validated antibodies. Please note that GE does not guarantee the performance of these antibodies, and the user must validate the antibodies conjugated at their site.

Required Equipment

Table 5: Summary table of the required equipment and how each is used in the workflow.

A	B
Equipment	Definitions
Timer	Used for timed reactions and/or processes
Humidified Chamber	Used for staining
Pipet boy	Used for serological pipettes
Pipettes (2-1000µL)	Deliver liquid volumes (µL) for dilutions and solutions
Staining dish	Slide reagent container used during incubations
Slide racks	Rack to hold slides in place during incubations
Orbital Shaker	Decoverslipping process and incubations
Analytical balance	Weighing out reagents
Microcentrifuge	Spinning down solutions in tubes
Vortexer	Mix solutions in tubes
Magnetic stir plate	Used for stirring solution
Spin bar	Used for mixing solutions on stir plate

SAFETY WARNINGS

Safety warnings and precautions
Warning: For research use only. Cell DIVE software and workflows are for internal research use only and not for third party service use or clinical diagnosis.
All chemicals should be considered as potentially hazardous. We therefore recommend that this product is handled only by those persons who have been trained in laboratory techniques and that it is used in accordance with the principles of good laboratory practice. Wear suitable protective clothing such as laboratory coats, safety glasses, and gloves. Care should be taken to avoid contact with skin or eyes. In the case of contact with skin or eyes wash immediately with water.

Please refer to the Safety Data Sheets (SDS) for health and environmental hazards.

BEFORE START INSTRUCTIONS

Reagent Prep

Working Solutions Table 1: Summary of the required working solutions for the workflow.

A	B
Working Solutions	Expiration
1X Phosphate Buffered Saline (PBS)	100 mL 10x PBS<br>900mL ddH20<br>----------------------<br>1000mL Total Volume<br><br>Mix thoroughly
Ab Diluent (3% BSA in PBS)	50 mL 1X PBS<br>1.5 g BSA<br>----------------------<br>50 mL Total Volume<br><br>Mix thoroughly and vortex in a 50 mL tube until all particulates are in solution. Store at 4C for no longer than a week.
DAPI Staining Solution	0.1 mL DAPI stock solution<br>499.9 mL 1X PBS<br>----------------------<br>500 mL Total Volume<br><br>Add 250 mL to 2 opaque staining dishes.<br>Discard after 10 uses. Working solution expires in 6 months from preparation. Store the solution in 4C.

A	B
Mounting Media	10 mL 1X PBS<br>90 mL glycerol<br>4.0 g Propyl Gallate<br>1.0 g DABCO<br>-----------------------<br>100 mL Total Volume<br><br>Mix contents in a glass bottle and heat overnight in water bath at 60C. Keep protected from light.<br><br>The next day make sure that all contents are in solution and filter with a 0.45uM filter. Cover with foil and store at 4C for up to 2 months.<br><br>Alternate mounting media (50% glycerol, 4% propyl gallate) should be used with markers that leach.
Dye Inactivation Solution	If using white coplin jars (hold ~50mL)<br>10 mL 0.5 M NaHCO3 , pH 11.2 (acceptable range 10.9-11.3)<br>35 mL ddH2O<br>5 mL 30% H2O2<br>------------<br>50 mL total volume<br><br>If using green staining jars (hold ~250mL) 50 mL 0.5 M NaHCO3 , pH 11.2 (acceptable range 10.9-11.3) 175 mL ddH2O 25 mL 30% H2O2<br>------------<br>250 mL total volume<br><br>Mix ddH2O and 0.5M NaHCO3 (pH 11.2) thoroughly. This makes up Part 1 of the solution. The 30% H2O2 is Part 2 of the solution.<br>Immediately before putting the slides in the solution, add Part 2 to Part 1 and mix thoroughly.

Critical Parameters

Please take particular note of the following instructions regarding critical steps:

- It is essential to read the complete instruction booklet before starting work.
- These instructions have only been validated on formalin-fixed paraffin embedded tissue sections.
- Unless noted, it is essential to allow reagents discussed to reach room temperature prior to use.
- Mix samples and all reagents thoroughly before use.
- The pH of the 0.5 M $NaHCO_3$ must be between pH 10.9 and pH 11.3 .
- Avoid extensive exposure of fluorescent reagents to ambient light.

Background Imaging

1 Refer to Cell DIVE Imaging Manual.

Decoverslipping

1h 55m

2

> **Note**
>
> Coverslips are removed from slides to prepare them for the next slide processing step.

3 Take a plastic slide box and place it in secondary containment.

4 Fill the plastic slide box with 1x PBS.

5 Rest the coverslipped slides on the interior raised edges of slide box such that they are inverted (ie. coverslip down and barcode up).

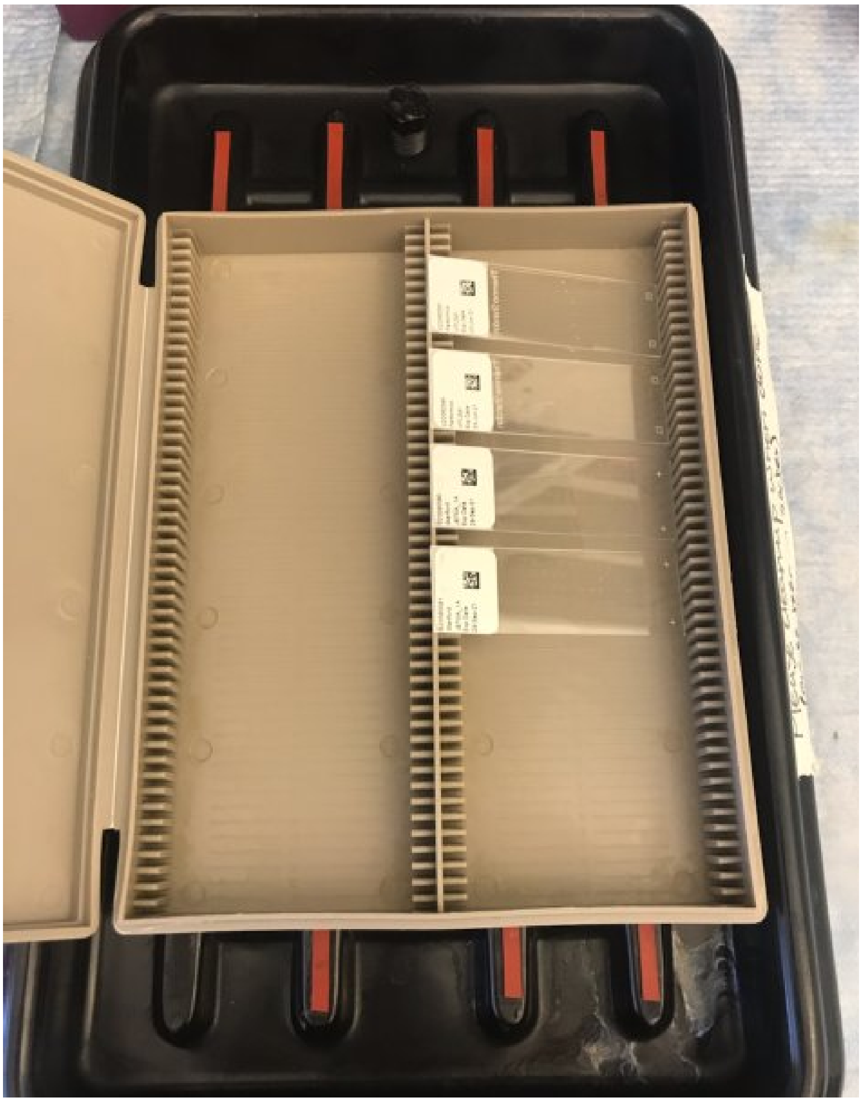

6 If your next round is a staining round, prepare your dilutions (see **Determining the Order of Antibodies for Staining** and **Calculations for Antibody Cocktails**) while you are waiting for the coverslips to come off.

7 Be patient - within 00:30:00 - 01:00:00 , coverslips should naturally come off. 1h 30m

Determining the Order of Antibodies for Staining

8 Below is a table of the segmentation markers available for use in the single cell analysis workflow. These markers are required to run single cell analysis.

A	B	C

A	B	C
Segmentation Marker	Required for SCA?	Segmentation used for
DAPI	Y	Nuclear
S6	Y	Cytoplasmic
NaKATPase	Y	Membrane
PCK26	Y	Epithelial

These segmentation markers are critical to the single cell analysis pipeline, and early staining of them is important to avoid the presence of any tissue damage or movement in the images, if the tissue is susceptible to either. That being said, the order of the segmentation markers relies on what other antibodies are in the study. The following rules are recommended for determining the order of antibodies for staining:

- If there are antibodies being used with primary/secondary detection, those must be stained prior to any direct conjugate raised in the same species.
- Antibodies that have antigen effects from dye inactivation should be stained in an early round.
- Once staining with the primary-secondary antibodies and the antibodies with antigen effects is completed, proceed onto the following segmentation markers
  - o S6
  - o NaK
  - o PCK26

Calculations for Antibody Cocktails

1h 35m

9

Note

The following section should be used when making cocktails of antibodies for any type of experiment including primary/secondary (P/S) antibody, directly conjugated (DC) antibody, or zenon-labelled antibody. For P/S antibodies, the species (Ex- rabbit, mouse IgG1) must be different to run on the same slide at the same time.

10 Determine the total volume that will be needed for the experiment.

- For manual staining (200µL*#of slides)

11 Determine the stock concentration for the antibodies. This should be written on the barcode or label on the antibody. If there is no concentration written on the vial or the datasheet, contact either the vendor and or chemist who prepared it and ask. Provide the catalog number and lot#, if applicable.

12 Once a concentration is obtained for an antibody, determine the final concentration the antibodies or conjugates should be run at.

- If the antibody/DC is new and commercially available and no final concentration is noted on the vendor

datasheet, call the vendor to ask. If no final concentration can be recommended, run the new marker at [M] 5 microgram per milliliter (µg/mL) .

- If the Ab is a GE Validated DC, refer to the DC catalog to determine the optimal working concentration.

13 Use the following formula to calculate the required amount for each antibody (Ab) -

- stock concentration*X=final concentration Ab1*total volume needed for experiment
- stock concentration*X=final concentration Ab2*total volume needed for experiment
- Solve for X in each case to determine how many µL of each antibody/DC will be required.
- Subtract the sum of the Ab volumes together from the total volume to get the volume of Ab diluent (3% BSA in PBS) that needs to be added.

Note

For example - if 20 µL of Ab1 and 40 µL of Ab2 was used, the sum of Ab volumes=60 µL. Subtract this volume from the total volume needed for the experiment to obtain the volume of diluent to add to the Abs.

14 If you are using secondary antibodies, determine the volume you will need for the secondary in the same manner described above for calculating for primary antibody/DC. Secondary antibodies are stained at a final concentration of [M] 5 microgram per milliliter (µg/mL) . Determine which secondaries you need based on the isotypes of the P/S antibodies used.

15 Determine if there is a dye preference for each marker (ie. Some markers look better in Cy3 or Cy5). If there is no preference, place the marker that has lower expression in Cy3 if possible.

Note

Check to make sure the dyes are different when making cocktails with DCs. For P/S antibodies, different species as well as different dyes must be used for each antibody. For example, a mouse IgG1 antibody and a rabbit IgG antibody could be pooled and an example of pooled secondary antibodies to use could be Cy3-Donkey anti-mouse and Cy5- Donkey anti-rabbit.

16 If you are staining with -

- primary/secondary antibodies, click below to proceed to **1°/2° Ab**.
- directly conjugated antibodies, click below to proceed to **Conjugated Ab**.
- zenon-labeled antibodies, click below to proceed to **Zenon Ab**.

Step 16 includes a Step case.

1°/2° Ab

Conjugated Ab

Zenon Ab

Ab Staining – Primary-Secondary Antibodies

2h 30m

step case

1°/2° Ab

17 Retrieve primary antibody from the 4 °C refrigerator or -20 °C freezer. **Be sure to keep On ice at all times.**

18 Spin the stock antibody down at 13000 x g, 00:01:00 . If stock antibodies are in large or odd shaped vials, transfer them to eppendorf tubes and label appropriately with a LIMS sticker (if possible, remove the LIMS label from the original vial and stick it on the eppendorf tube).

19 Make up primary antibody dilutions in the antibody diluent solution ([M] 3 % (v/v) BSA in 1x PBS) using the manufacturer suggested concentration of antibody, with the exceptions below.

- If there is no concentration suggested, then use [M] 5 microgram per milliliter (µg/mL)
- If the vendor suggests < [M] 1 microgram per milliliter (µg/mL) , then increase it to [M] 1 microgram per milliliter (µg/mL) (when performing 1 hour room temperature incubation).
- If no concentration or recommended dilution is available, use a 1:100 dilution.

20 Spin down dilutions at 13000 x g, 00:01:00 and keep dilutions On ice . Put antibodies back to proper storage location after making up dilutions.

21 Slides should be in 1X PBS (from decoverslipping step); remove from PBS and turn slide on its side and gently tap slide on blue pad/Kimwipe to remove solution. Remove as much of the PBS solution without letting the tissue dry out and without touching the tissue.

22 Place slides in humidified chamber. Chamber should contain water in the bottom. Move quickly in adding the primary antibody so that slides do not dry out.

23 Add a total of 200 µL antibody dilution to each slide; cover with parafilm cut to a size just larger than tissue area. Make sure there are no bubbles under the parafilm. Place lid on humidified chamber.

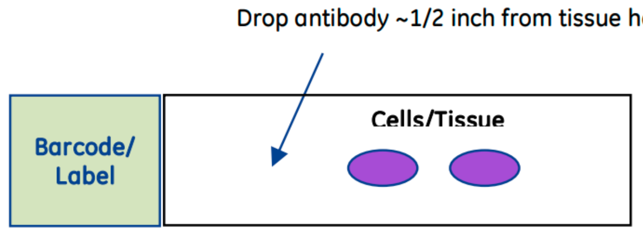

24 Incubate for 01:00:00 at Room temperature . 1h

25 Retrieve secondary antibody aliquot from -20 °C ; keep On ice and then spin down at 13000 x g, 00:01:00 .

26 Make up secondary antibody dilutions in the antibody diluent solution ([M] 3 % (v/v) BSA in 1x PBS). Secondary antibodies are stained at a final concentration of [M] 5 microgram per milliliter (µg/mL) .

27 Spin down dilutions at 13000 x g, 00:01:00 . Keep On ice in light protected environment while the primary antibody incubation is taking place.

28 Remove parafilm and place slides in coplin jar or staining dish. Wash slides 3x at 5 minutes each with 1X PBS, using gentle agitation on shaker:

28.1 (Wash 1/3) Wash slides for 00:05:00 with 1X PBS, using gentle agitation on shaker. 5m

28.2 (Wash 2/3) Wash slides for 00:05:00 with 1X PBS, using gentle agitation on shaker. 5m

28.3 (Wash 3/3) Wash slides for 00:05:00 with 1X PBS, using gentle agitation on shaker. 5m

29 After final PBS wash, turn slide on its side and gently tap slide on blue pad/Kimwipe to remove remaining PBS. Once again remove as much of the liquid without letting the tissue dry out and without touching the tissue.

30 Place slides in humidified chamber. Add a total of 200 µL secondary antibody dilution to each slide just as suggested above. Cover with parafilm cut to a size just larger than tissue area. Make sure there are no bubbles under the parafilm. Place lid on humidified chamber.

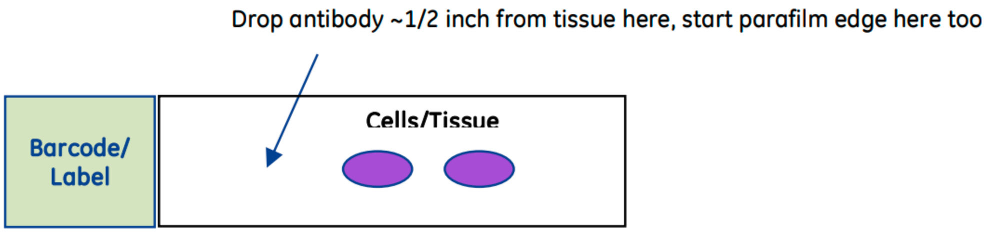

31 Incubate for 01:00:00 at Room temperature. 1h

32 Remove parafilm and place slides in coplin jar or staining dish. Wash slides 3x at 5 minutes each with 1X PBS, using gentle agitation on shaker:

32.1 (Wash 1/3) Wash slides for 00:05:00 with 1X PBS, using gentle agitation on shaker. 5m

32.2 (Wash 2/3) Wash slides for 00:05:00 with 1X PBS, using gentle agitation on shaker. 5m

32.3 (Wash 3/3) Wash slides for 00:05:00 with 1X PBS, using gentle agitation on shaker. 5m

Note

A 2 minute DAPI recharge is recommended but not required after staining.

33 Proceed *immediately* to **Coverslipping**.

Coverslipping

34

Note

Slides are coverslipped to protect the tissue from drying out.

35 Mounting media should be removed from the 4 °C fridge and brought to Room temperature .

36 Slides should be quickly dipped in ddH2O.

37 Take a rainin pipet tip (200uL-green box) and with a scissors clip the end to make a wide bore.

38 Take up 75 µL mounting media (4% propyl gallate, 1% DABCO, 90% glycerol) and add to one end of the slide. For leaching markers, use alternate mounting media (50% glycerol, 4% propyl gallate).

39 Place the end of the coverslip at a 45 degree angle and slowly lower; allowing the mounting media to flow under the coverslip across the slide.

40 Make sure there is not mounting media oozing out of the sides of the coverslip; remove any excess by gently blotting on a Kimwipe.

41 Store the slides at 4 °C in light protected environment. When ready for imaging, proceed to **Imaging**. Recommendation: image within 24 hours of staining.

Imaging

42 Proceed to **Imaging** as per the Cell DIVE^{TM} Imaging manual.

- If you are imaging a stain, you are conducting a stain round.
- If you are imaging post-dye inactivation, you are conducting a background round.

43 After imaging, you will proceed on to either dye inactivation or staining.

- If you just imaged your background round and are ready to stain, proceed to **Decoverslipping** and then **Determining the Order of Antibodies for Staining**.
- If you just imaged your stain ready and are ready to dye inactivate, proceed to Section **Decoverslipping** and then **Dye Inactivation**.

Dye Inactivation

1h 4m

44

Note

Dye inactivation is performed after every staining round, except for the last round. Dye inactivation rounds vary for each stain based on the bleaching efficiency, but the majority of rounds will need just 1x of dye inactivation post-stain. (PCK26 and AE1 require 2-3 rounds of dye inactivation.)

45

Note

The acceptable range for the pH of the 0.5 M $NaHCO_3$ is between pH 10.9 and pH 11.3. Check and log the pH on a weekly basis during periods of use. If the pH is measured outside of this specification, discard the buffer and do not use.

Refer to the antibody catalogue to understand which antibodies have antigen effects with this dye inactivation method. Use this information to design your multiplexing experiment such that the antigens sensitive to dye inactivation are stained first.

46 Fill a “PBS” labeled staining dish with fresh 1X PBS. Place decoverslipped slides to a slide rack and transfer to this staining dish.

47 Transfer Dye Inactivation Solution into a “Dye Inactivation” labeled staining dish.

Note

Refer to the working solution for dye inactivation. The hydrogen peroxide is added to the solution immediately before adding to the staining dish. This reaction is time sensitive.

48 Transfer the slide rack into “Dye Inactivation” staining dish and incubate for 00:15:00 at Room temperature . 15m

49 If more than one round is needed, following each dye inactivation incubation/round, transfer slide rack into staining dish containing fresh 1X PBS for 00:01:00 . Prepare a fresh batch of Dye Inactivation Solution for the next round during this one minute wash. 1m

50 Once the dye inactivation rounds are complete, wash the slides 3X with fresh 1X PBS for 5 minutes each with gentle agitation on an orbital shaker set to 60 RPM:

50.1 (Wash 1/3) Wash slides for 00:05:00 with 1X PBS, using gentle agitation on shaker set to 60 RPM. 5m

50.2 (Wash 2/3) Wash slides for 00:05:00 with 1X PBS, using gentle agitation on shaker set to 60 RPM. 5m

50.3 (Wash 3/3) Wash slides for 00:05:00 with 1X PBS, using gentle agitation on shaker set to 60 RPM. 5m

51 DAPI signal diminishes after dye inactivation. Slides are re-stained with DAPI to label the cell nuclei for imaging purposes.

52 Prepare the DAPI Staining Solution.

53 Pour the DAPI Staining Solution into a “DAPI” labeled staining dish.

54 Transfer the slide rack sitting in 1X PBS into the "DAPI" staining dish.

55 Incubate slides in DAPI Staining Solution for 00:02:00 at Room temperature with gentle agitation on an orbital shaker set to 60 RPM. 2m

56 After the 2 minute incubation, transfer slide rack into a staining dish containing fresh 1X PBS.

Note

DO NOT DISCARD DAPI; the DAPI may be reused up to 10 times or by the expiration date, whichever comes first. Tally the number of uses on the DAPI bottle

57 Wash slides for 00:05:00 in 1X PBS with gentle agitation on an orbital shaker set to 60 RPM. 5m

58 Proceed *immediately* to **Coverslipping** and then **Imaging**.
