# Supplementary material for: Pathobiology of Candida auris infection analyzed by multiplexed imaging and single cell analysis
Source: PLoS One. 2024 Jan 17;19(1):e0293011. doi: 10.1371/journal.pone.0293011 (PMC10793899; doi:10.1371/journal.pone.0293011)
Supplement: S3 File — Protocol document from protocol io (dx.doi.org/10.17504/protocols.io.bpwumpew), which outlines slide clearing and antigen retrieval procedures to prepare slides for multiplex imaging. (PDF) [file pone.0293011.s003.pdf]

DEC 10, 2020

Cell DIVE™ Platform | Slide Clearing and Antigen Retrieval

In 1 collection

Liz McDonough[1], Chrystal Chadwick[1], Fiona Ginty[1], Christine Surrette[1], Anup Sood[1]

[1]GE Research

Human BioMolecular Atlas Program (HuBMAP) Method Development Community | GE F

Liz McDonough
GE Research

OPEN ACCESS

DOI: dx.doi.org/10.17504/protocols.io.bpwumpew

Protocol Citation: Liz McDonough, Chrystal Chadwick, Fiona Ginty, Christine Surrette, Anup Sood 2020. Cell DIVE™ Platform | Slide Clearing and Antigen Retrieval. **protocols.io** https://dx.doi.org/10.17504/protocols.io.bpwumpew

License: This is an open access protocol distributed under the terms of the Creative Commons Attribution License, which permits unrestricted use, distribution, and reproduction in any medium, provided the original author and source are credited

Protocol status: Working
We use this protocol and it's working

Created: Nov 20, 2020

Last Modified: Dec 10, 2020

PROTOCOL integer ID: 44724

Keywords: Cell DIVE™ Platform Slide Clearing, platform slide clearing, slide clearing, deparaffinization, rehydration, Cell DIVE

ABSTRACT

The purpose of this protocol is to manually deparaffinize and rehydrate slides for the Cell DIVE™ Platform.

ATTACHMENTS

Cell_DIVE-manual_slide-clearing_final_version.pdf

GUIDELINES

Definitions

A	B
Definition/Acronym	Definitions
BSA	Bovine Serum Albumin
PBS	Phosphate Buffered Saline
ARS	Antigen Retrieval Solution
AR	Antigen Retrieval
RT	Room Temperature (18 to 25oC)
dH2O	Distilled water
FFPE	Formalin-fixed, paraffin embedded
RPM	Rotations per minute
DC	Direct Conjugate
PPE	Personal Protective Equipment
DC	Direct Conjugate
RV	Revalidation

Descriptions

Paraffin embedded formalin fixed slides have to be cleared first to (1) remove the wax out with xylene and (2) rehydrate the tissue back into buffer with a series of ethanol, etc. steps. After clearing, formalin bridges have to be uncrossed by incubating the tissue slides in a pressure cooker with antigen retrieval solutions. This document covers the slide clearing and antigen retrieval process using manual protocol.

Troubleshooting

A	B	C
Issue	Potential Cause	Next Steps
If tissue fall off or floats off after clearing	Fixation protocol might not be longer<br><br>Or slide type is not positively charged<br><br>Or paraffin used during embedding is soft	Please contact vendor/collaborator<br><br>Mount tissue on positive charged slides<br><br>Please contact vendor or GE tech support and ask about baking overnight at 60C option instead of 1 hour as per the procedure
Wax on the slide after clearing	Xylene wash not enough	Please contact GE tech support.<br>If necessary repeat clearing<br>protocol with additional xylene<br>washes
Tissue falls off after antigen retrieval	Tissue is susceptible to heat and pressure	Please consult GE tech support<br>for next steps or alternate AR<br>solution.
DAPI staining did not work	DAPI contaminated or tissue problem	Make sure to re-DAPI with freshly made DAPI. If problem<br>continues, contact GE tech support.

MATERIALS

Required Materials

A	B
Material	Definitions
0.45µm filter	Filtration of mounting media
Amber glass bottles	Storage for mounting media and/or DAPI
Amber 1.5mL Eppendorf tubes	Storage for antibody direct conjugates
15mL conical tubes	Storage for mixing solutions
50mL conical tubes	Storage for Antibody diluent

A	B
Weigh Boats	Used to weigh out solid/powder reagents on analytical balance
Leica Coverslips	Used for Leica coverslipper
Eppendorf 1.5mL tubes	Container for antibody dilution preparations
Pipette Tips (0.5-1000µL)	Used for pipettes
Serological Pipettes	Deliver liquid volumes (mL) for solutions
Transfer Pipettes	Delivers PBS for decoverslipping
Lab Tape	Labels bottles and tally scoring
Xylene-Resistant slide labels	Labels printed on the Zebra printer which are resistant to xylene
Nitrile Gloves	Personal Protective Equipment
Kimwipes (large and small)	Clean mounting media from slides
Blue Underpads	Underpad used to absorb reagent spills on the work bench

Table 1: Summary of required materials

Required Reagents and Stock Solutions

A	B
Stock Solutions	Expiration
Donkey Serum	Reconstitute the donkey serum with 10 mL of dd H20. Store in -20 deg C.
10x Tris Antigen Retrieval Solution	12.1 g 10x Tris<br>3.7 g EDTA<br>5.0 mL Tween 20<br>995 mL ddH20<br>------------------------<br>1000mL Total Volume<br><br>Place in beaker with stir bar and mix thoroughly for 10 minutes<br>pH the solution to 9<br><br>Aliquot into (20) 5 mL and (36) 25 mL aliquots. Barcode all aliquots with cryolabels. All aliquots should have the<br>same barcode/label with the date.<br><br>Store in -20 deg C for no longer than 6 months.
10% Triton X-100	10 mL Triton X-100<br>90 mL ddH20<br>------------------------<br>100 mL Total Volume<br>Mix thoroughly

A	B
DAPI Stock Solution	10 mg vial of DAPI dilactate<br>2.0 mL ddH20<br>---------------------<br>2 mL Total Volume<br><br>Mix thoroughly until solid is dissolved.<br><br>Aliquot into (20) 100 uL aliquots into amber eppendorfs.<br><br>Store in -20 deg C.

Table 2: Summary of the required stock solutions and reagents for the workflow.

Expirations and storage conditions for stock reagents and solutions

A	B	C
Solutions	Expiration	Storage
Liquid Chemicals		
Stock Citrate Antigen Retrieval Solution (Unmasking Solution)	6 months from receipt	4 degrees
10x Tris Antigen Retrieval Solution, pH 9.0	6 months from receipt	-20
Ethanol	2 years from receipt	RT-flammable cabinet
DABCO 4mM	1 week	4 degrees
Phosphate buffered saline	1 year from receipt	RT
Tween	2 years from receipt	RT
DAPI	1 year from receipt	-20
Dry Chemicals		
Propyl gallate	2 years from receipt	RT
DABCO	2 years from receipt	4 degrees
BSA	2 years from receipt	4 degrees
EDTA	2 years from receipt	RT
Donkey Serum	1 year from receipt	-20
Sodium Azide	2 years from receipt	RT

Table 3: Summary of the required reagents and stock solutions, and their respective shelf time and storage conditions.

Required Equipment

A	B
Equipment	Definitions
Analytical balance	Weighing out reagents

A	B
Black Leica slide rack	Rack to hold slides in place in the Leica Autostainer XL and Leica Coverslipper instruments
Blue slide rack	Rack to hold slides in place during incubations
Decloaker	Antigen retrieval process
Graduated Cylinders (25 mL, 100 mL, 250 mL, 500 mL, 1000 mL)	Used to measure solution volumes
Gray slide rack	Rack to hold slides in place during antigen retrieval and incubations
Gripper Apparatus	Holds slides in place during decoverslipping process
Humidified Chamber	Used for phosphatase pretreatment
Incubator	Slide baking process
Metal staining dish	Slide reagent container used in the decloaker instrument
Microcentrifuge	Spinning down solutions in tubes
Orbital Shaker	Decoverslipping process and incubations
pH meter	Measuring pH of dye inactivation/bleaching solution, ARS1 and ARS2
Pipet boy	Used for serological pipettes
Pipettes (2-1000µL)	Deliver liquid volumes (µL) for dilutions and solutions
Staining dish	Slide reagent container used during incubations
Stir plate	Mixing solutions
Timer	Used to time reactions and/or processes
Vortexer	Mix solutions in tubes

Table 4: Summary table of the required equipment and how each is used in the workflow.

Working Solutions

A	B
Working Solutions	Expiration
1X Phosphate Buffered Saline (PBS)	100 mL 10x PBS<br>900mL ddH20<br>------------------------<br>1000mL Total Volume<br><br>Mix thoroughly
95% Ethanol	950 mL 200 proof Ethanol<br>50mL ddH20<br>------------------------<br>1000mL Total Volume<br><br>Mix thoroughly

A	B
70% Ethanol	700 mL 200 proof Ethanol<br>300mL ddH20<br>----------------------<br>1000mL Total Volume<br><br>Mix thoroughly
50% Ethanol	500 mL 200 proof Ethanol<br>500mL ddH20<br>----------------------<br>1000mL Total Volume<br><br>Mix thoroughly
0.3% Triton X-100, 1XPBS	13.5 mL 10% Triton<br>436.5 mL 1X PBS<br>----------------------<br>450 mL Total Volume<br><br>Mix thoroughly
Citrate Antigen Retrieval Solution (ARS1)	If you're using white coplin jars (50 mLs) -<br>2.5 mL Stock Antigen Unmasking Soln (Vector Labs H3300)<br>47.5 mL ddH20<br>----------------------<br>50 mL Total Volume<br><br>If you're using green staining jars (hold ~250 mL)<br>12.5 mL Stock Antigen Unmasking Soln (Vector Labs H3300)<br>237.5 mL ddH20<br>----------------------<br>250 mL Total Volume<br><br>Shake stock unmasking solution vigorously prior to pipetting. Mix water and stock unmasking solution thoroughly in beaker. Make fresh prior to each antigen retrieval step. The pH should be 6.0
Tris Antigen Retrieval Solution (ARS2)	If you're using white coplin jars (50 mLs) -<br>5 mL 10x Tris AR stock<br>45 mL ddH20<br>----------------------<br>50 mL Total Volume<br><br>If you're using green staining jars (hold ~250 mL)<br>25 mL 10x Tris AR stock<br>225 mL ddH20<br>----------------------<br>250 mL Total Volume<br><br>Mix water and stock Tris AR thoroughly in beaker.<br>Make fresh prior to each antigen retrieval step.<br>The pH should be 8.8-9.0
Slide Blocking Solution (10% donkey serum, 3% BSA, 1xPBS)	20 mL reconstituted donkey serum<br>180 mL 1X PBS<br>6.0 g BSA<br>----------------------<br>200 mL Total Volume<br><br>Mix thoroughly
DAPI Staining Solution	0.1 mL DAPI stock solution<br>499.9 mL 1X PBS<br>----------------------<br>500 mL Total Volume<br><br>Add 250 mL to 2 opaque staining dishes.<br>Discard after 10 uses. Working solution expires in 6 months from<br>preparation.

A	B
Mounting Media	10 mL 1X PBS<br>90 mL glycerol<br>4.0 g Propyl Gallate<br>1.0 g DABCO<br>------------------------<br>100 mL Total Volume<br><br>Mix contents in a glass bottle and heat overnight in water bath at 60C. Keep protected from light.<br><br>The next day make sure that all contents are in solution and filter with a 0.45uM filter. Cover with foil and store at 4C for up to 2 months.<br><br>Alternate mounting media (50% glycerol, 4% propyl gallate) should be used with markers that leach.

Table 5: Summary of the required working solutions for the workflow.

A	B	C
Working Solutions	Expiration	Storage
Citrate Antigen Retrieval Solution (ARS1)	Daily: Make fresh prior to antigen retrieval step	N/A
Tris Antigen Retrieval Solution (ARS2)	Daily: Make fresh prior to antigen retrieval step	N/A
Dapi	6 months from making	4 degrees

Table 6: Summary of the required working solutions for the workflow.

SAFETY WARNINGS

Warning: For research use only.

Cell DIVE software and workflows are for internal research use only and not for third party service use or clinical diagnosis.

All chemicals should be considered as potentially hazardous. We therefore recommend that this product is handled only by those persons who have been trained in laboratory techniques and that it is used in accordance with the principles of good laboratory practice. Wear suitable protective clothing such as laboratory coats, safety glasses, and gloves. Care should be taken to avoid contact with skin or eyes. In the case of contact with skin or eyes wash immediately with water.

BEFORE START INSTRUCTIONS

Please take particular note of the following instructions regarding critical steps:

- It is essential to read the complete instruction booklet before starting work.
- These instructions have only been validated on formalin-fixed paraffin embedded tissue sections.
- Unless noted, it is essential to allow reagents discussed to reach room temperature prior to use.
- Mix samples and all reagents thoroughly before use.
- Avoid extensive exposure of fluorescent or light sensitive reagents to ambient light.

Slide Baking

1

Note

This is the initial step of the deparaffinization process. This step serves two purposes: (1) melting of the paraffin wax and (2) ensuring tissue adherence to the slides.

2 Set the incubator to 60 °C .

3 Load tissue slides into a slide rack. All tissue slides should have the tissue side facing up and parallel to the rack.

Note

For fragile/fatty tissues, the slides may be placed in front of a fan overnight, before slide baking, to remove excess moisture.

4 Place slide rack (with tissue side facing up) into a 60 °C incubator.

5 Incubate for at least 01:00:00 . We recommend Overnight baking for optimal tissue retention.

Reagent Preparation

6 After placing FFPE slides in the incubator for baking, prepare the following reagents – xylene, 100% ethanol, 95% ethanol, 70% ethanol, 50% ethanol, 1X PBS, and 1X PBS with 0.3% Triton X100.

Note

Reagent amounts (250 mL) are for a staining dish that can contain up to 24 slides.

Note

If only 5 slides, use a coplin jar and make up all of the working solutions to a 50 mL final volume.

Note

Make sure to fill up the container only up to frost edge (lower edge). Avoid pouring solutions all the way up to the top of the slide (labels and pen writing on frosted edge may contaminate samples).

7 Before preparing reagents, place blue underpads on the bench to absorb spills.

8 After baking, follow steps below.

9 Transfer slides to xylene and wash, 00:05:00 each, with gentle agitation (1/2). 5m

10 Transfer slides to xylene and wash, 00:05:00 each, with gentle agitation (2/2). 5m

11 Place slides in 100% ethanol and wash, 00:05:00 each with gentle agitation (1/2). 5m

12 Place slides in 100% ethanol and wash, 00:05:00 each with gentle agitation (2/2). 5m

13 Place slides in 95% ethanol and wash, 00:05:00 each with gentle agitation (1/2). 5m

14 Place slides in 95% ethanol and wash, 00:05:00 each with gentle agitation (2/2). 5m

15 Place slides in 70% ethanol and wash, 00:05:00 each with gentle agitation (1/2). 5m

16 Place slides in 70% ethanol and wash, 00:05:00 each with gentle agitation (2/2). 5m

17 Place slides in 50% ethanol and wash, 00:05:00 each with gentle agitation (1/2). 5m

18 Place slides in 50% ethanol and wash, 00:05:00 each with gentle agitation (2/2). 5m

19 Place slides in PBS and wash for 00:05:00 each with gentle agitation (1/2). 5m

20 Place slides in PBS and wash for 00:05:00 each with gentle agitation (2/2). 5m

21 Permeabilize tissue in 1X PBS with 0.3% Triton X100 for 00:10:00 . 10m

22 Wash in 1X PBS for 00:05:00 . 5m

Note

During this wash, begin pre-warming the antigen retrieval solutions that are required for the next step. Refer to the "Antigen Retrieval" Section for additional information regarding solution prep.

Antigen Retrieval

23 During slide clearing and hydration prepare the reagents needed for antigen retrieval (ARS1+ARS2):

23.1 Remove a 25 mL aliquot of the 10x Tris Antigen Retrieval Solution (ARS2) from the -20°C freezer.

23.2 Allow the ARS2 to thaw at Room temperature .

24 Prepare ARS1 (Citrate based solution):

24.1 Label a beaker or 250ml cylinder “ARS1”.

24.2 Add 237.5 mL ddH20 to the beaker.

24.3 Remove Vector Labs citrate antigen retrieval solution from 4°C storage.

24.4 Shake citrate solution vigorously.

24.5 Add 12.5 mL citrate solution to ARS1 beaker.

24.6 Add a magnetic stir bar, place on magnetic stirrer, and stir thoroughly (if using a graduated cylinder, cover with parafilm and invert to mix).

24.7 Transfer to a metal staining dish from the NxGen Decloaker labeled "ARS1."

24.8 pH using Biocare pH strips, verify pH is 6.0.

25 Prepare ARS2 (Tris based solution).

25.1 Label a beaker or 250ml cylinder "ARS2".

25.2 Add 225 mL ddH20 to the beaker.

25.3 Once the aliquot of 10x ARS2 has thawed, add it to the beaker. If the aliquot is partially thawed, you can

pour the frozen solution into the water.

25.4 Add a magnetic stir bar, place on magnetic stirrer, and stir thoroughly. If using a graduated cylinder, cover with parafilm and invert to mix.

25.5 Transfer to a metal staining dish from the NxGen Decloaker labeled “ARS2.”

25.6 pH using Biocare pH strips, verify pH is between pH 8.5 and pH 9.

26 Prepare the Biocare NxGen Decloaker.

26.1 Add 500 mL DI water to the bottom of pressure cooker.

26.2 Be sure to add fresh DI water before each antigen retrieval run.

27 Fill 3 metal staining dishes (provided with the Biocare NxGen decloaker) containing the two antigen retrieval solutions (ARS1 and ARS2) and DI water (250 mL).

Note

The maximum amount of slides per run is 24.

Note

pH - Citrate should be 6 and TRIS should be 8.5/9.

28 Place the staining dishes containing the antigen retrieval solutions in the wire rack in the outside positions and the staining dish containing the water in the center position.

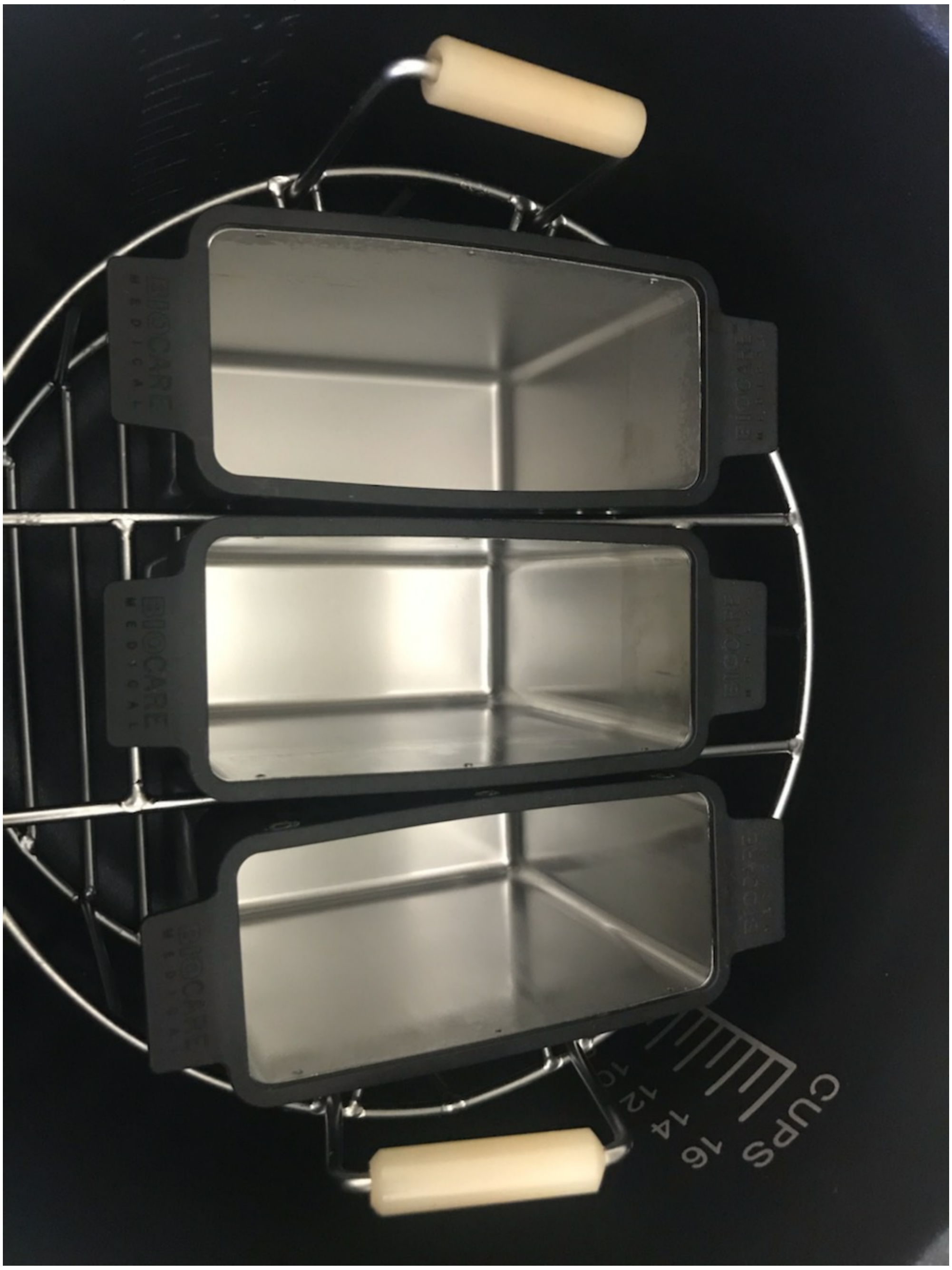

29 Place the wire rack with the staining dishes into the water bath.

30 Plug the decloaker into a wall outlet to turn the unit on – it is not equipped with a power switch. The display screen will turn on.

31 Press the lower left hand button (Menu button: page with lines) once and a list of temperatures will appear. Select 🌡 110 °C using the menu button (lower left) and press the green circle (center button) once.

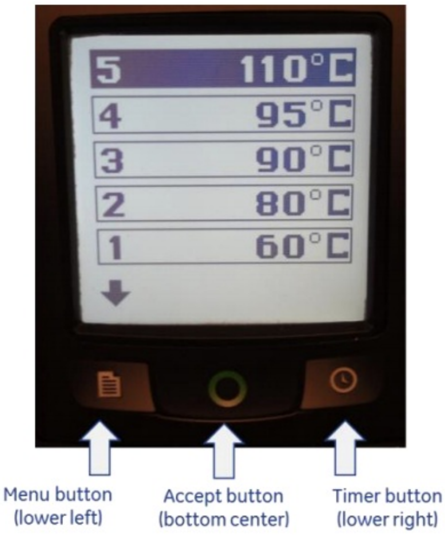

32 Set the time to ⏱ 00:04:00 using the lower menu and timer buttons as indicated on the screen and press the green button to accept. 4m

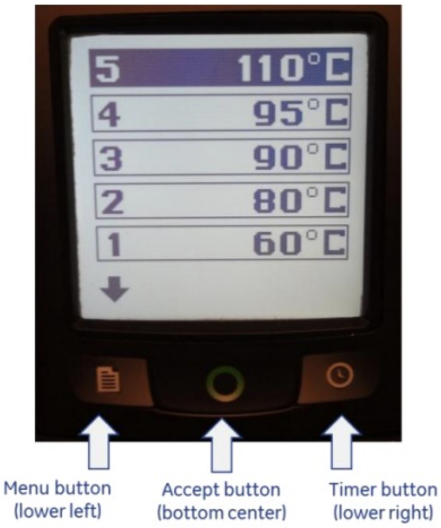

33 The pressure cooker is now waiting to start and can sit in this state until the slides are ready.

34 Complete the below checklist and include it in your documentation for the Antigen Retrieval run.

A	B	C
Pre-Warming Antigen Retrieval Checklist	Duration (min)	Comments
Added 500mL of fresh DI water to the pressure cooker?	5 minutes	
ARS1 and ARS2 prepared appropriately?		Consult working solutions section.
pH'd both ARS1 and ARS2 prior to heating?		ARS1 pH should be 6, ARS2 pH should be 8.5/9.

35 Place slides in the Citrate-Antigen Retrieval working solution (ARS1). Place steam test strip on the center staining (water) dish so that it rests across the top of the staining dish without touching liquid. Please note that the metal staining dishes do not have lids.

36 Place the lid on the Decloaking chamber and make sure it is in the locked position (metal button over the left handle); turn lid towards you to lock. Position the pressure limit valve as directed in image below, ensuring it is set level on the pressure stem, not tipped to one side.

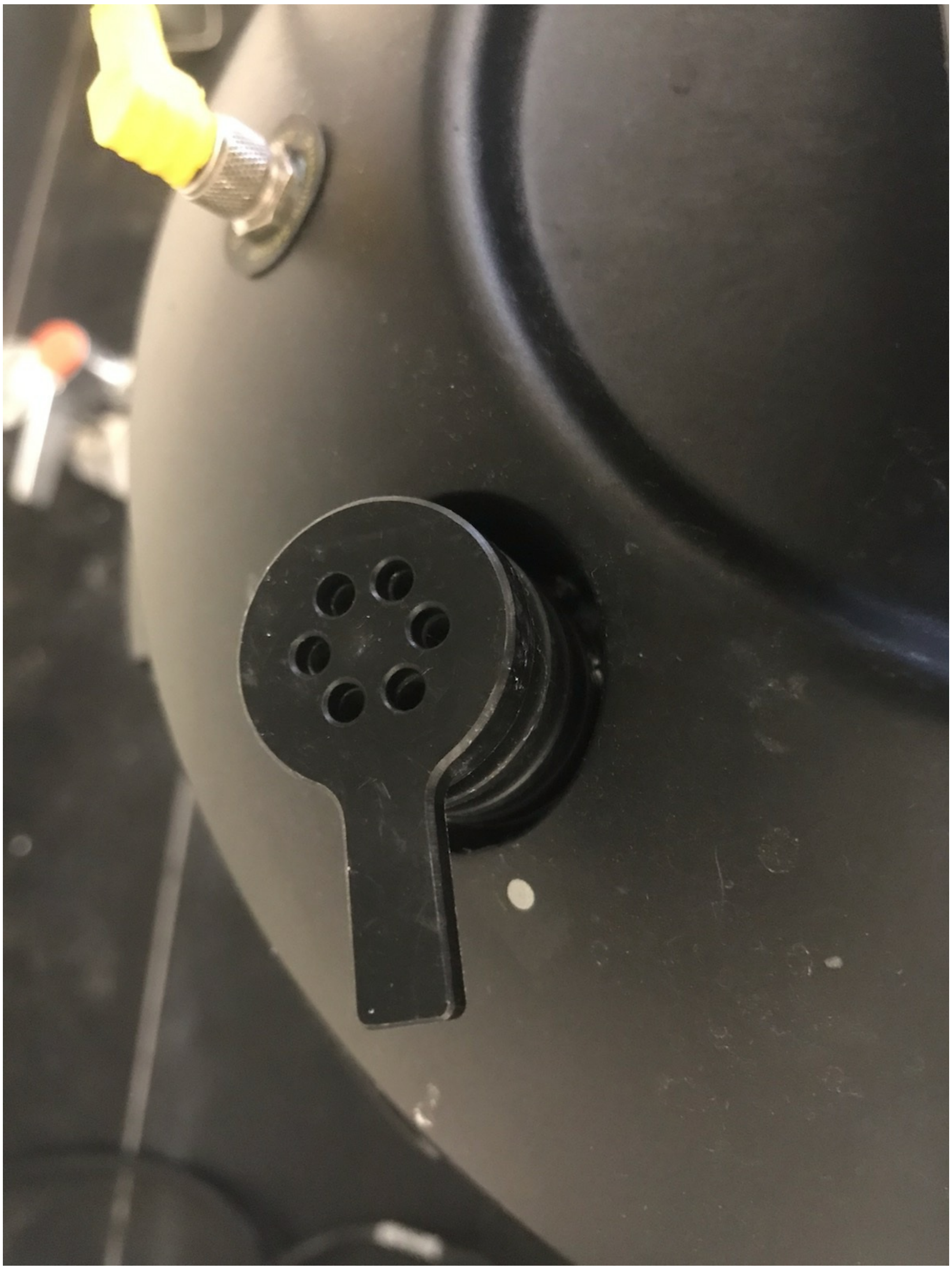

37 Press the center green button once to begin the program. When the temperature display on the front of the decloaker shows 70 °C start an external timer for 00:20:00 . 20m

Note

The slides will spend 20 minutes in ARS1, 20 minutes in ARS2, and then sit for 10 minutes on the benchtop in ARS2. The program will count down 4 minutes once the pressure cooker has reached the set temperature of 110°C. The external timer is used to time the 20 minutes required in ARS1 since the program does not do this.

38 Pressure cooker will hold at 110 °C for 00:04:00. Once the 4 min hold is complete, record the temperature and pressure from the device display on the log sheet. Leave the slides in ARS1 solution for a total time of 00:20:00 (until the external timer beeps). Over the remaining time, the temperature should decrease. 24m

39 After the external timer has beeped, record the displayed temperature and pressure on the log sheet.

40 Carefully open the lid (use gloves as the surface is hot). Water will run from the lid when tipped so be sure to not allow the water to pour into either of the AR solutions. Remove steam strip indicator, steam strip should be uniformly brown if passed (light brown is OK). Allow steam strip to dry and then tape into log sheet.

41 Transfer the slides into Tris-Antigen Retrieval working solution (ARS2) and close pressure cooker (it will not lock after the run is complete, but it will slowly cool down even though it is not in the locked position).

42 Restart the external timer for 00:20:00. During the 20 minutes, the decloaker will beep to indicate the run has been completed. Press the green circle (center button) to stop the beeping, but leave the decloaker closed and slides in ARS2. The temperature will decrease during this time. 20m

43 After the external timer has beeped, open pressure cooker, and record the final displayed temperature and pressure on the log sheet.

44 Check pH of solutions with test strips and record on log sheet. For the pH, AR solution 1 (citrate) should be pH 6 and AR solution 2 (Tris) should be pH 8.5 / pH 9.

45 Remove the wire rack and place on the bench for 00:10:00 (keep slides in hot Tris-ARS2 during this cool-down). 10m

46 Let the pressure cooker cool down to 50 °C. Unplug the pressure cooker to turn it off.

47 Wash slides (00:05:00 each) in PBS with gentle agitation on shaker (1/4). 5m

48 Wash slides (00:05:00 each) in PBS with gentle agitation on shaker (2/4). 5m

49 Wash slides (00:05:00 each) in PBS with gentle agitation on shaker (3/4). 5m

50 Wash slides (00:05:00 each) in PBS with gentle agitation on shaker (4/4). 5m

51 Proceed to slide blocking.

Slide Blocking

52 During slide clearing and hydration prepare the reagents needed for slide blocking.

52.1 Remove two 10 mL aliquots of reconstituted donkey serum from the -20°C freezer.

52.2 Allow the donkey serum to thaw at Room temperature .

53 Prepare Blocking Solution. Let the slides sit in 1X PBS until the blocking solution is ready.

53.1 Label a beaker “Blocking Solution.”

53.2 Add 180 mL 1x PBS to the beaker.

53.3 Weigh out and add 6.0 g Bovine Serum Albumin (BSA) to the beaker.

53.4 Add a magnetic stir bar, place on magnetic stirrer, and stir thoroughly.

53.5 While stirring, add the 2x 10 mL aliquots of donkey serum to the beaker.

53.6 Continue to mix thoroughly.

53.7 Cover with parafilm and leave at Room temperature until you are ready for slide blocking.

54 Once you’re ready for slide blocking, pour the prepared Slide Blocking Solution into a staining dish and transfer slides from 1X PBS into the Slide Blocking staining dish.

55 Incubate for at least 01:00:00 at Room temperature, or Overnight at 4 °C. 1h

56 After the incubation, wash slides one time with fresh 1X PBS for 00:05:00 on an orbital shaker set to 60 RPM. 5m

Initial DAPI Staining

57 If DAPI working solution is not available, prepare it as per Table 5 in the "Reagent Prep" Section.

58 Pour DAPI solution into a staining dish.

59 Transfer blue slide rack (containing the slides) sitting in 1X PBS into the “DAPI” staining dish.

> Note
>
> FFPEs are stained with DAPI to label the cell nuclei for imaging purposes.

60 Incubate slides in DAPI working solution for 00:15:00 at Room temperature with gentle agitation on an orbital shaker set to 60 RPM. 15m

61 After the 15 minute incubation, transfer the slide rack into a staining dish containing fresh 1X PBS.

> Note
>
> *Do not discard the DAPI solution.* The DAPI may be reused up to 10 times or by the expiration date, whichever comes first. Tally the number of uses on the DAPI bottle.

62 Wash slides 1x for 00:05:00 in fresh 1X PBS with gentle agitation on an orbital shaker set to 60 RPM. 5m

63 Transfer slides to staining dish with dH_20 and proceed to coverslipping.

Coverslipping Slides

64 Mounting media should be removed from the 4°C fridge and brought to Room temperature .

65 Take a rainin pipet tip (200uL-green box) and with a scissors clip the end to make a wide bore.

66 Take up 50 µL mounting media (4% propyl gallate, 1% DABCO, 90% glycerol) and add to one end of the slide. For leeching markers, use alternate mounting media (50% glycerol, 4% propyl gallate).

67 Place the end of the coverslip at a 45 degree angle and slowly lower; allowing the mounting media to flow under the coverslip across the slide.

68 Check to make sure there are no bubbles over the tissue. Also make sure there is not mounting media oozing out of the sides of the coverslip; remove any excess.

69 Proceed to Background Imaging or store the slides at 4 °C in light protected environment.
