# Supplementary material for: Pathobiology of Candida auris infection analyzed by multiplexed imaging and single cell analysis
Source: PLoS One. 2024 Jan 17;19(1):e0293011. doi: 10.1371/journal.pone.0293011 (PMC10793899; doi:10.1371/journal.pone.0293011)
Supplement: Attachment — Submitted filename: Response-to-Reviewers.docx [file pone.0293011.s004.docx]

Nicholas A. Pullen, Ph.D.
Academic Editor
PLOS ONE

September 15, 2023

Dr. Nicholas A. Pullen and PLOS ONE editorial staff:

In response to academic editor and reviewer responses regarding our original manuscript (PONE-D-23-02779) originally entitled “Pathobiology of *Candida auris* infection analysed by multiplexed imaging and single cell analysis”, by Chrystal Chadwick, Magdia De Jesus, Steven R. Torres, Fiona Ginty, and Jessica S. Martinez, has been revised. We have outlined each point of concern and annotated how each was addressed in our revised manuscript.

1.) We have changed the manuscript title to: Pathobiology of *Candida auris* infection **analyzed** by multiplexed imaging and single cell analysis. This change in our revised manuscript is also noted in our cover letter.
2.) We have **removed author Steven R. Torres** as he does not meet authorship metric outlined by PLOS One for this revised manuscript and has not contributed to the drafting of our revised manuscript. In the acknowledgements we as mentioned the funding source for Dr. Magdia De Jesus to be start-up funds provided by University at Albany and Wadsworth Center. We have stated these changes in our cover letter.
3.) In our cover letter, we also provided our “Competing Interests Statement” to say, **“The authors have no existing competing interests.”**

We are also providing a rebuttal letter that responds to each point raised by the academic editor and reviewer(s) and uploaded this letter as a separate file labeled 'Response to Reviewers' as instructed.

As instructed, a marked-up copy of our manuscript that highlights changes made to the original version has also been uploaded as a separate file labeled 'Revised Manuscript with Track Changes'.

We are also providing an unmarked version of our revised paper without tracked changes and uploaded as a separate file labeled 'Manuscript'.

4.) We have also made our raw data for Figures 2-7 available through a stable repository. Data is provided here <https://doi.org/10.5281/zenodo.8144269>, where we provide in a zip file, the OME files used in multiplex single cells analysis. These OME files can be open with QuPath as described in the materials and methods section.

**Line 200-202**: “The images used in this analysis are provided in stable repository: <https://doi.org/10.5281/zenodo.8144269>. Images in the repository are provided as a zipfile, were the OME files can be downloaded and opened with QuPath for further analysis.”

5.) We have provided protocols as supporting information S1-S3 and in protocols.io as follows:

**Lines 122-127:** “…Protocols are also described in protocols.io: Cell DIVE™ Platform Slide Clearing and Antigen Retrieval ([dx.doi.org/10.17504/protocols.io.bpwumpew](https://dx.doi.org/10.17504/protocols.io.bpwumpew)), Cell DIVE™ Platform Antibody Characterization for Multiplexing ([dx.doi.org/10.17504/protocols.io.bpyxmpxn](https://dx.doi.org/10.17504/protocols.io.bpyxmpxn)), and [Cell DIVE™ Platform Antibody Staining & Imaging(](https://www.protocols.io/view/cell-dive-platform-antibody-staining-amp-imaging-rm7vz3b22gx1/v1)[dx.doi.org/10.17504/protocols.io.bpv6mn9e](https://dx.doi.org/10.17504/protocols.io.bpv6mn9e))…”

6.) We have ensured our manuscript meets PLOS ONE's style requirements, including those for file naming.
7.) We have completed our “Competing Interests Statement” to say, **“The authors have no existing competing interests.”** and we also mentioned this in our cover letter.
8.) We have ensured our data and protocols have been made available and would like to change our “Data Availability” statement to reflect this update. **We have made our data available through Zenodo, a stable repository for data,** and protocols through protocol io as follows: Cell DIVE™ Platform Slide Clearing and Antigen Retrieval ([dx.doi.org/10.17504/protocols.io.bpwumpew](https://dx.doi.org/10.17504/protocols.io.bpwumpew)), Cell DIVE™ Platform Antibody Characterization for Multiplexing ([dx.doi.org/10.17504/protocols.io.bpyxmpxn](https://dx.doi.org/10.17504/protocols.io.bpyxmpxn)), and [Cell DIVE™ Platform Antibody Staining & Imaging(](https://www.protocols.io/view/cell-dive-platform-antibody-staining-amp-imaging-rm7vz3b22gx1/v1)[dx.doi.org/10.17504/protocols.io.bpv6mn9e](https://dx.doi.org/10.17504/protocols.io.bpv6mn9e). We mention this change regarding our repository information in our cover letter.
9.) Supporting information captions have been included at the end of our manuscript and cited in **Lines 122-127**.
10.) Reference list has been reviewed and ensured to be complete and correct.

**Reviewer #1:**

1.) However the current submission appears to be hurried up (non-curated bibliography, poor description of statistical methods for quantification, Fig. 5 is the same as Fig. 7 [two duplicated brain figures but no heart one for me to review], poor resolution images) and the work would benefit from a thorough revision.

*Our apologies to PLOS ONE editor and reviewers for having uploaded two figures of brain (duplicate mistake with Fig 5.) This error was due to an upload mix-up on our end during the original submission process. We have corrected this mistake and have uploaded a Fig 5 showing data for heart. We have also improved resolution of images and processed them through the Preflight Analysis and Conversion Engine (PACE) digital diagnostic tool to ensure all figures meet PLOS requirements.*

*We have also done a through editorial revision of the manuscript to correct any grammatical mistakes and provide further clarity throughout the article.*

2.) In introduction on line 64 use reference 15 after the term MxIF is introduced for the first time. So the original work of Gerdes et al., 2013 PNAS 110(29):11982-7 is referenced first. This work appears twice in the references list: 15 and 27. Please make sure that all the references are correct and numbered appropriately before resubmission.

*Reference list has been updated and corrected.*

3.) line 66: is it not "60+" too colloquial?

*We used the description in previous publications. We prefer to maintain it for consistency with our other publications involving our outlined multiplex technology.*

4.) line 103: "scarified sacrificed". Please correct to "sacrificed".

***Line 103..now Line 101-102****. Corrected.*

*“…Histological slides utilized in this study were from mice were sacrificed on day 7 after C. auris infection…”*

5.) lines 124-129: reference supplementary material or add those web links to the references if allowed by the journal format. You did that for Image J and QuPath.

*Supporting information captions have been included at the of our manuscript and cited in* ***Lines 122-127****.*

*“…Slides were cleared and processed for antigen retrieval as previously described(15).* *Protocols (see S1-S3 fig) are also described in protocols.io: Cell DIVE™ Platform Slide Clearing and Antigen Retrieval (*[*dx.doi.org/10.17504/protocols.io.bpwumpew*](https://dx.doi.org/10.17504/protocols.io.bpwumpew)*), Cell DIVE™ Platform Antibody Characterization for Multiplexing (*[*dx.doi.org/10.17504/protocols.io.bpyxmpxn*](https://dx.doi.org/10.17504/protocols.io.bpyxmpxn)*), and* [*Cell DIVE™ Platform Antibody Staining & Imaging(*](https://www.protocols.io/view/cell-dive-platform-antibody-staining-amp-imaging-rm7vz3b22gx1/v1)[*dx.doi.org/10.17504/protocols.io.bpv6mn9e*](https://dx.doi.org/10.17504/protocols.io.bpv6mn9e)*)...”*

6.) line 135: There is a double full stop.

***Line 135..now Line 133.*** *Corrected.*

*“…After blocking, slides were stained with DAPI (1 µg/ml), cover-slipped with anti-fade mounting media, and background images collected in DAPI, FITC, Cy3 and Cy5 filter channels to record native autofluorescence (“background acquisition”).”*

7.) line 170: You can just reference (Table 2) and eliminate this "Ly6G and CD45R/B220 at 10 ug/mL, CD45, CD3, and CD68 at 5 ug/mL" The concentrations are listed in Tab.2.

***Line 170…now Line 169.*** *Corrected.*

*“…Antibodies used in this study were found to be suitable for MxIF analysis at manufacturer's recommended concentrations, see Table 2...”*

8.) Statistical analysis, plots and figures: It is not clear how many abscess were counted and how many serial sections per organ were analyzed. The quantification raw data should be part of supplementary material.

*We outline how serial sections per organ were analyzed in Line 134-202. To summarize, “…the whole tissue [was] imaged for DAPI, followed by image stitching to create a composite image of the sample…A minimum of 9 regions with >10 fields were chosen for each histological section, with fungal abscesses serving at the target histological criterial for use in ROI selection throughout various tissue sections of multiple organs… A minimum of 10 ROIs were used to measure…ratio to total immune cell…”*

*To better clarify, we added to Lines 147-148: “…Analysis in this study was conducted with single serial sections for each organ type to demonstrate feasibly of MxIF for fungal infections.”*

*In Lines 183-202 we also specify our method for calculating abscess area and our statistical analysis. To summarize: “…The log base 10 of abscess area was calculated…to analyze size distribution of fungal abscess…quantity of abscesses was measured as a count ratio of abscesses within infected tissues to uninfected control groups…**Unpaired T-test was used to determine statistically significant differences between groups…”*

*We have provided the OME image files for each organ in a stable repository, so readers of this manuscript can have high resolution images and be able to conduct the same analysis described using ImageJ or QuPath.*

9.) Could you please use an overlay of scatter and box plot so that the reader could see the individual data? The area graphs lack a unit of measure which I assume is um^2. If possible you could conceive combining Fig. 2-4 in one picture with letter panels. I could not review Fig. 5 which was supposed to present heart data as Fig. 7 (brain) was accidentally included twice.

*As we have addressed in reviewers first comment, we have corrected the mistake with Fig 5. We have also improved resolution of images and processed them through the Preflight Analysis and Conversion Engine (PACE) digital diagnostic tool to ensure all figures meet PLOS requirements.*

*Graphs in Figures 2-4 measuring fungal abscess area show log base 10. We clarified in Lines 183-187 the size of abscesses area: “The log base 10 of abscess area* ***(µm^2^ in size)*** *was calculated and graphed using a box and whisker plot to analyze size distribution of fungal abscess for wildtype infected mice (non-neutropenic) and infected neutropenic mice (1A8 and RB6 treated) within each organ tissue.”*

*Although an insightful suggestion by the reviewer to combine Figures 2-4, we believe keeping figures separate, highlights organ specific analysis.*

10.) The resolution of the images is extremely poor, it would be nice for the reviewer to have access to better ones. For Fig 5-7 it would be beneficial to use letter panels.

*Figures used in this manuscript processed through the Preflight Analysis and Conversion Engine (PACE) digital diagnostic tool to ensure all figures meet PLOS requirements. In addition, we have also made images for Figures 2-7 available through a stable repository.*

*Data is provided here* [*https://doi.org/10.5281/zenodo.8144269*](https://doi.org/10.5281/zenodo.8144269)*, where we provide in a zipfile, the OME files used in multiplex single cells analysis. These OME files can be open with QuPath or ImageJ, providing readers with images of the highest resolution possible for this study.*

11.) line 375: "andindependent". Include space.

***Line 375..now Line 376.*** *Corrected.*

*“…We also found the presence of B cells to be few and independent of neutropenia, which further supports studies that have shown C. auris does not elicit an IgG driven B cell response (39)…”*

12.) line 410: "CD3 dominate". Correct to "CD3-dominant"

***Line 410.*** *Corrected.*

*“…Overall, we found organ specific immune niches within heart, kidney, and brain tissues infected with C. auris, revealing the heterogenous distribution of fungi and a neutrophil driven immune response, which shifts toward a CD3-dominant response in the absence of neutrophils, sustaining abundant and persistent abscesses within infected tissues…”*

13.) line 434-436: Remove the abbreviations for the organisms mentioned (e.g. Acinetobacter baumannii (AB)) as they are not used elsewhere in the manuscript.

***Line 434-436…now Line 434-439.*** *Corrected.*

*“…**Increased prevalence of co-infections with of multi-drug resistant fungal organisms (e.g. C. auris) with viruses (e.g. COVID-19), and gram-negative bacteria (e.g. Acinetobacter baumannii , Pseudomonas aeruginosa, and carbapenem resistant enterobacteria, like Klebsiella pneumoniae), (8–10) pose a fatal risk to immunocompromised patient populations and warrants a better of fungal pathobiology. …”*

14.) Also the period starting at 432 reads a little odd. Consider rephrasing. I would also mention Carbapenem Resistant Enterobacterales instead of the sole K. pneumoniae.

*We agree with reviewer 1 feedback. We have edited* ***Line 434-439*** *as follows:*

*“…Increased prevalence of co-infections with of multi-drug resistant fungal organisms (e.g. C. auris) with viruses (e.g. COVID-19), and gram-negative bacteria (e.g. Acinetobacter baumannii , Pseudomonas aeruginosa, and carbapenem resistant enterobacteria, like Klebsiella pneumoniae), (8–10) pose a fatal risk to immunocompromised patient populations and warrants a better of fungal pathobiology…”*

**Reviewer #2:**

1.) Given that this paper is classified as a methods paper, suggest providing a bit more context about previous usage of MxIF or similar methods have been used for C. auris or other related yeast, both in mouse models or other.

*To the knowledge of authors in this manuscript there have not been multiplex imaging studies conducted with C. auris. Our efforts were to demonstrate feasibility of MxIF for use in analyzing fungal infection pathology at the single cell level. We did however, in Lines 62-79 and Lines 387-409, demonstrate the use of MxIF and how application in fungal diagnostics may provide more insight into its pathology and potential aid in development of therapeutics.*

2.) One interesting point is that a C. ablicans antibody was used that is cross-reactive with C. auris. Can the authors comment on any implications or limitations this cross-reactivity and whether a C. auris specific antibiody is available or could be of value?

*In Lines 207-212 we describe the use of a polyclonal antibody against C. albicans (Thermo Fisher Cat No. PA1-7206), which was found to be cross-react with C. auris by Torres, S.R. and team in their 2020 publication.*

*To the awareness of the authors in this manuscript, there is no commercially available antibody for C. auris, and we do believe it would be of value, especially in future MxIF analysis. We have included this comment in the revised manuscript: “…No commercially available antibody against C. auris was identified for use in this study and future MxIF analysis would benefit from such a resource.”*

3.) Along similar lines, I think table 2 should clarify that the C. auris target antibody is actually marketted as a C. albicans antibody. Even though this is indicated elsewhere in the text, I think some readers may not catch this so ideal if authors can find a way to incorporate this into the table.

*We agree with reviewer’s insightful feedback. We have amended Table 2 to clarify the antibody used to image/stain for C. auris was conducted with a Thermo Fisher antibody marketed for its cross-reactivity with C. albicans.*

4.) Line 51 states that CDC has identified 3-5% of all isolates globally are pan-resistant to antifungals, but the associated reference (Ref #3) is not by CDC authors and does not seem relevant. Suggest correcting reference.

*We disagree with the reviewer that reference 3 is not relevant. Reference 3 does summarize CDC findings and list numerous CDC references regarding pan-resistance of C. auris against multiple medical fungicides.*

*To better clarify our reference to this publication, we removed the direct mention of the CDC, to emphasize that the listed reference does mention the multi-drug resistance evolution of C. auris. See Lines 49-52: “.**C. auris has become a global public health challenge and* *3-5% of isolates identified world-wide, exhibit pan-resistance that includes resistance to the azoles, polyenes and most importantly the echinocandins, a third class of antifungals currently used to clinically treat resistant* *C. auris (3).”*
