# Supplementary material for: Pathobiology of Candida auris infection analyzed by multiplexed imaging and single cell analysis
Source: PLoS One. 2024 Jan 17;19(1):e0293011. doi: 10.1371/journal.pone.0293011 (PMC10793899; doi:10.1371/journal.pone.0293011)
Supplement: Attachment — Submitted filename: Response-to-Reviewers.docx [file pone.0293011.s005.docx]

Ashok Kumar, Ph.D.
Academic Editor
PLOS ONE

September 29, 2023

Dr. Ashok Kumar and PLOS ONE editorial staff:

In response to academic editor and reviewer responses regarding our revised manuscript (PONE-D-23-02779R1) entitled “Pathobiology of *Candida auris* infection analyzed by multiplexed imaging and single cell analysis”, by Chrystal Chadwick, Magdia De Jesus, Steven R. Torres, Fiona Ginty, and Jessica S. Martinez, has been further edited. We have outlined each point of concern and annotated how each was addressed in our second revision.

1.) We have made changes to all the minor grammatical/writing corrections outlined by Reviewer 3.

Line 55: eliminate ‘carries’—was removed.
2) Line 102: eliminate ‘were’ before sacrificed—was removed.
3) Lines 115, 272, 285, 297, 309 and any other places: correct to Ly6G and not LyG6—corrected.
4) Line 149: add a comma between primary secondary. —was added.
5) Line 199: replace ‘were’ with ‘where’—replaced.
6) Line 431: better understanding of.. —line has been corrected.
7) Make sure to write the scientific names in italics throughout the manuscript. One example is in lines 209, 220, 314 etc. —corrected.

2.) Figures 5, 6, and 7 were also revised to provide clear images and we have included larger composite images. Right composite images should all biomarkers, while the left only shows those for immune cells used in the study. Color was also changed for B220 and Ly6G biomarkers and revision was reflected in the figure captions.

We also reviewed references and entire manuscript to ensure it is complete and correct. Our revised figures 5-7 were also reviewed through the Preflight Analysis and Conversion Engine (PACE) digital diagnostic tool to ensure they meet PLOS requirements. We hope you find our second revised manuscript to meet PLOS ONE publication criteria and look forward to your response.

Sincerely,

Dr. Jessica Martinez; Lead Scientist

Biosciences, GE Research

One Research Circle Niskayuna, NY 12309

Office: 518-387-4506; Mobile:  518-386-9566

E-mail: [jessica.s.martinez@ge.com](mailto:jessica.s.martinez@ge.com)
